# Long noncoding antisense RNA FAM83A‐AS1 promotes lung cancer cell progression by increasing FAM83A

**DOI:** 10.1002/jcb.28336

**Published:** 2019-01-18

**Authors:** Rongxing Shi, Zichen Jiao, Ao Yu, Tao Wang

**Affiliations:** ^1^ Department of Cardiothoracic Surgery Nanjing Drum Tower Hospital, Nanjing University Medical School Nanjing China; ^2^ Department of Cardiothoracic Surgery Nanjing Drum Tower Hospital, Nanjing Medical University Nanjing China

**Keywords:** FAM83A, FAM83A‐AS1, long noncoding RNA, natural antisense transcripts, non–small‐cell lung cancer

## Abstract

The abnormal expression of long noncoding RNAs (lncRNAs) is closely associated with human cancers. As one special group of lncRNAs, natural antisense transcripts (NATs) can be transcribed from both DNA strands at the same locus but in the opposite direction from the gene transcript. Their expression levels are altered in many cancers, but their roles are poorly understood. We strove to find NATs involved in human non–small‐cell lung cancer (NSCLC) and to reveal their mechanism of action in cancer. We analysed the NATs in NSCLC from the TCGA database by circlncRNAnet. One NAT, family with sequence similarity 83 member A antisense RNA 1 (FAM83A‐AS1), was found to be markedly upregulated and positively correlated with its cognate sense counterpart, FAM83A, in NSCLC. Moreover, overexpression of FAM83A‐AS1 increased FAM38A protein levels and induced ERK1/2 phosphorylation downstream of FAM83A in cells. Finally, overexpression of FAM83A‐AS1 promoted LUAD cell proliferation and invasion. In summary, lncRNA FAM83A‐AS1 promotes LUAD by increasing FAM83A expression.

## 1 INTRODUCTION

The incidence of lung cancer has dramatically increased in the last ten years, with more than 1.6 million new patients diagnosed each year; lung cancer is the most common malignant tumour.^1^ Small cell lung carcinoma and non–small‐cell lung carcinoma (NSCLC) are the main types of lung cancer. NSCLC is the most common malignant lung tumour worldwide, with extremely high morbidity and mortality rates.^1^ The two most common subtypes of NSCLC are lung adenocarcinoma (LUAD) and lung squamous cell carcinoma (LUSC). LUAD accounts for 40% of all lung cancer cases, whereas LUSC accounts for 30%. At present, the main treatment of NSCLC is still surgery. Despite the progress in medical and surgical treatments for NSCLC in recent years, the overall survival time of patients with NSCLC has not changed substantially, and the 5‐year survival rate is still approximately 15%.^1^ Therefore, NSCLC requires the identification of new therapeutic targets via discovering the mechanisms underlying NSCLC progression.

Natural antisense transcript RNA (NAT), a specific long noncoding RNA, refers to a transcript that binds to a specific DNA or RNA.^2^ It is an endogenous species that exists in the living organism under natural conditions and is produced in the opposite direction to the transcription of the target transcript. In humans, NATs account for approximately 22% of the entire genome. An increasing number of studies has highlighted a link between NATs and cancers.^3^ However, the biological signiﬁcance of NATs remains under scientiﬁc investigation, with major questions yet to be answered.

In this study, we focused on NATs in NSCLC. First, we analysed the NATs in LUAD and LUSC from the TCGA database by circlncRNAnet, which identified 11 NATs with marked and consistent upregulation in both LUAD and LUSC. We further confirmed these 11 NATs in five LUAD tissues and five LUSC tissues and found that one NAT, family with sequence similarity 83 member A antisense RNA 1 (FAM38A‐AS1), was markedly upregulated and positively correlated with its cognate sense counterpart, FAM83A, in cancer tissues. Then, we found that overexpression of FAM83A‐AS1 increases FAM38A protein levels and activates its downstream signalling pathway in A549 cells. Finally, overexpression of FAM83A‐AS1 promoted LUAD cell proliferation and invasion. The current ﬁndings suggest that long noncoding RNA (lncRNA) FAM83A‐AS1 could increase FAM83A expression and promote carcinogenesis.

## 2 MATERIALS AND METHODS

### 2.1 Human tissue samples

All methods and experimental protocols were approved by Nanjing University and carried out in accordance with the corresponding guidelines. The biospecimens were provided by Nanjing Multicentre Biobank, the biobank of Nanjing Drum Tower Hospital, the Affiliated Hospital of Nanjing University Medical School, with the informed consent of every donor, and a normalized ethnic audit was performed. The tissue specimens used in this study were frozen in liquid nitrogen immediately after dissection and stored at −80°C. Each tissue specimen was verified histologically and pathologically by a pathologist, and the results are listed in Table 1.

**Table 1:** Patients' characteristics

Case nos	Age	Sex	Pathological stage	Pathological type
#1	55	M	II	LUAD
#2	42	F	I	LUAD
#3	65	M	II	LUAD
#4	48	M	II	LUAD
#5	57	F	II	LUAD
#6	61	M	III	LUSC
#7	56	F	II	LUSC
#8	39	F	III	LUSC
#9	44	M	I	LUSC
#10	60	F	II	LUSC

Abbreviations: LUAD, lung adenocarcinoma; LUSC, lung squamous cell carcinoma.

### 2.2 Cell culture

The human alveolar adenocarcinoma cell line A549 was cultured in Dulbecco's modified Eagle's medium (DMEM), fortified with 10% fetal bovine serum (FBS; GIBCO, NY) at 37°C in a 5% CO_2_ humidified atmosphere.

### 2.3 Online database analysis

The circlncRNAnet database (http://app.cgu.edu.tw/circlnc/)^4^ [42] was utilized to analyse the NATs in LUAD and LUSC. To explore the expression levels of FAM38A‐AS1 and FAM38A, we downloaded the RNA‐Seq raw data and survival data of patients with LUAD and LUSC from the TCGA data portal (http://cancergenome.nih.gov/).

### 2.4 Quantitative real‐time PCR

Total RNA from the frozen tissue specimens and cultured cells was isolated with TRIzol reagent (Invitrogen, Carlsbad, CA) according to the manufacturer's instructions. To quantify the NATs, RT products including SYBR Green (TAKARA, Dalian, China) and primers designed for the NATs and glyceraldehyde 3‐phosphate dehydrogenase (GAPDH) were utilized. The primer sequences were as follows: FOXD3‐AS1 (forward primer): 5′‐TCTGGCCTCAGTGCTCATTC‐3′;

FOXD3‐AS1 (reverse primer): 5′‐ACCTGAGTGGTTTGGTTGGG‐3′; FAM83A‐AS1 (forward primer): 5′‐CCCCAGAGCACTTCCTTAGC‐3′; FAM83A‐AS1 (reverse primer): 5′‐CAGGGCCGTCTGTGTTTACT‐3′; FEZF1‐AS1 (forward primer): 5′‐AGGGGATCGACGAGTTGAGA‐3′; FEZF1‐AS1 (reverse primer): 5′‐TTGTCCCCGAGTCATTGGTG‐3′; BARX1‐AS1 (forward primer): 5′‐AGAAGTGTCCCCAGGAGGTT‐3′; BARX1‐AS1 (reverse primer): 5′‐AGGAGGTAGGCCCTGTGATT‐3′; NOVA1‐AS1 (forward primer): 5′‐GCGCTGGTAGGCAGACTAAA‐3′; NOVA1‐AS1 (reverse primer): 5′‐GTGTTGCAGGGTTGACGTTC‐3′; POU6F2‐AS2 (forward primer): 5′‐GCCTGGCACCTAGAATTTG‐3′; POU6F2‐AS2 (reverse primer): 5′‐GGCAGGAAAGGGCACTGTTA‐3′; NPSR1‐AS1 (forward primer): 5′‐TGTTGAGAAGTGCACGGTCC‐3′; NPSR1‐AS1 (reverse primer): 5′‐GGCATGTGGTGACTATGCCA‐3′; BBOX 1‐AS1 (forward primer): 5′‐TGCAACTCCAAACCTAACG‐3′; BBOX 1‐AS1 (reverse primer): 5′‐GAGTGACTGGGGTCAGGGTA‐3′; KCNMB2‐AS1 (forward primer): 5′‐AACAGTGTGGGTCAGCCTT‐3′; KCNMB2‐AS1 (reverse primer): 5′‐TTGCCGTTTGACAGTTGTGC‐3′; ZFPM2‐AS1 (forward primer): 5′‐CCCTGAAGGCTTCTGCGATTA‐3′; ZFPM2‐AS1 (reverse primer): 5′‐TCTCCCTGGGTTACCACATGA‐3′; and GAPDH (forward primer): 5′‐CTGGGCTACACTGAGCACC‐3′; GAPDH (reverse primer): 5′‐AAGTGGTCGTTGAGGGCAATG‐3′.

### 2.5 Protein isolation and Western blot analysis

The frozen tissue specimens and cultured cells were lysed in RIPA lysis buffer with freshly added protease inhibitor cocktail (Roche, Mannheim, Germany) and prepared for Western blot analysis according to the manufacturer's instructions.^5^ Proteins were separated by 10% SDS‐PAGE before being electrotransferred to a PVDF membrane (Roche, Indianapolis, IN). The membrane was incubated with primary antibodies after 1 hour of blocking in 5% skim milk. The antibodies used were as follows: the anti‐FAM38A antibody (ab128245) and anti‐GAPDH antibody (EPR16891) were from Abcam (Cambridge, UK), and the antibodies against ERK1/2 and phospho‐ERK1/2 (Thr202/Tyr204) were from Cell Signaling Biotechnology. The signal was detected after treatment with SuperSignal West Pico chemiluminescence (Pierce, NIH). Protein bands were quantified using the ImageJ software (NIH).

### 2.6 Cell proliferation assay

The relative cell number was evaluated using the Cell Counting Kit‐8 (Dojindo) according to the manufacturer's instructions.^5^ Briefly, A549 cells were seeded in 96‐well plates at a density of 10^4^ cells per well and counted at the indicated time points after transfection with an empty vector or the FAM38A‐AS1 vector. A total of 10 μL of CCK‐8 reagent was added to each test well and incubated for 1 hour at 37°C. The absorbance was detected at a wavelength of 450 nm.

### 2.7 Cell migration assay

The migration of A549 cells was tested using a two‐chamber Transwell cell migration assay (BD Biosciences, MA) following the manufacturer's instructions. Briefly, the cells were harvested 24 hours after transfection and added to the upper chamber (10^4^ cells per well) with FBS‐free DMEM culture medium. At the same time, 0.5 mL of DMEM with 20% FBS was added to the lower compartment. The cells were incubated at 5% CO_2_ and 37°C for 18 hours. Then, cells on the upper surface of the Transwell chamber were removed by cotton swabs, and the migrated cells were fixed with 4% paraformaldehyde for 10 minutes and stained with crystal violet solution (0.5% in methanol) for 20 minutes at room temperature. The lower surfaces (containing the cells that had migrated) were imaged using a photomicroscope (×10 fields per chamber; BX51; Olympus, Japan).

### 2.8 Statistical analysis

All experiments were performed in triplicate, and each experiment was repeated at least three times. The data (mean ± SEM) are representative of at least three independent experiments. The numerical data were statistically analysed by the two‐tailed Student *t* tests. Statistical significance was defined as *P* < 0.05.

## 3 RESULTS

### 3.1 Identification of upregulated NATs in NCSC

**Figure 1:**
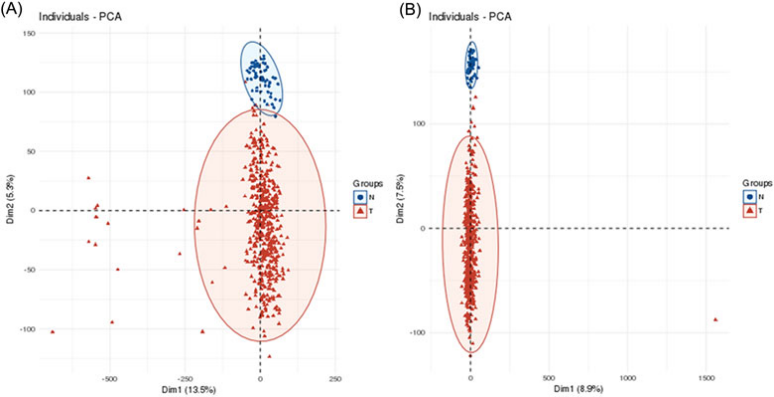
Principal component analysis showed that the tumour and normal samples were distinctly clustered by their lncRNA expression levels in LUAD (A), and LUSC (B). lncRNA, long noncoding RNA; LUAD; lung adenocarcinoma; LUSC, lung squamous cell carcinoma

**Figure 2:**
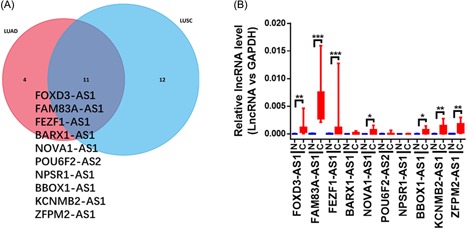
The upregulated NATs in LUAD and LUSC. (A) The upregulated NATs in LUAD and LUSC from the circlncRNAnet database. (B) The expression levels of FOXD3‐AS1, FAM83A‐AS1, FEZF1‐AS1, BARX1‐AS1, NOVA1‐AS1, POU6F2‐AS2, NPSR1‐AS1, BBOX 1‐AS1, KCNMB2‐AS1, and ZFPM2‐AS1 in five LUAD tissues and five LUSC tissues by qRT‐PCR. *P < 0.05; **P < 0.01; ***P < 0.001. LUAD, lung adenocarcinoma; LUSC, lung squamous cell carcinoma; NAT, natural antisense transcript; qRT‐PCR, quantitative real‐time PCR

First, we utilized circlncRNAnet to analyse the lncRNAs in LUAD and LUSC. Principal component analysis (PCA) showed that the tumour and normal samples were distinctly clustered according to their lncRNA expression levels in LUAD (Figure 1A) and LUSC (Figure 1B). Among these dysregulated lncRNAs, we found 405 dysregulated NATs in LUAD (322 NATs upregulated, 83 NATs downregulated; Supporting Information Table 1) and 444 dysregulated NATs in LUSC (286 NATs upregulated, 158 NATs downregulated; Supporting Information Table 2). For the upregulated NATs, filtering using a log 2 fold change > 5 and an FDR *P* < 0.05 identified 15 NATs in LUAD and 23 NATs in LUSC. From these NATs, we obtained 10 NATs (FOXD3‐AS1, FAM83A‐AS1, FEZF1‐AS1, BARX1‐AS1, NOVA1‐AS1, POU6F2‐AS2, NPSR1‐AS1, BBOX 1‐AS1, KCNMB2‐AS1, ZFPM2‐AS1) that were upregulated in both LUAD and LUSC (Figure 2A). We then proceeded to validate the expression levels of these 10 NATs in 10 paired normal tissues and cancer tissues from patients with NSCLC by qRT‐PCR analyses. The results showed that seven NATs were significantly upregulated in these patients, particularly FAM83A‐AS1 (Figure 2B). Because FAM83A‐AS1 was the most overexpressed AS in cancer tissues (Figure 2B), we chose FAM83A‐AS1 for further studies.

**Table 2:** The upregulated NATs in LUAD and LUSC

Genes	ENSG	Gene_full_name	LUAD		LUSC	
			Log 2 fold change	*p*adj	Log 2 fold change	*p*adj
FOXD3‐AS1	ENSG00000230798	FOXD3 antisense RNA 1 (head to head)	6.80	1.26E−68	7.52	9.98E−94
FAM83A‐AS1	ENSG00000204949	FAM83A antisense RNA 1	6.46	1.09E−117	6.30	2.56E−73
FEZF1‐AS1	ENSG00000230316	FEZF1 antisense RNA 1	6.07	9.92E−94	5.91	9.19E−85
BARX1‐AS1	ENSG00000235601	BARX1 antisense RNA 1 (head to head)	6.06	7.93E−30	6.56	4.28E−48
NOVA1‐AS1	ENSG00000257842	NOVA1 antisense RNA 1 (head to head)	5.66	1.96E−27	5.35	1.45E‐24
HOXC13‐AS	ENSG00000249641	HOXC13 antisense RNA	5.63	1.40E−27	8.23	8.62E−91
POU6F2‐AS2	ENSG00000233854	POU6F2 antisense RNA 2	5.54	1.01E−19	7.45	1.72E−91
NPSR1‐AS1	ENSG00000197085	NPSR1 antisense RNA 1	5.50	2.06E−41	6.68	1.24E−64
BBOX 1‐AS1	ENSG00000254560	BBOX 1 antisense RNA 1	5.48	5.31E−61	6.81	1.82E−206
KCNMB2‐AS1	ENSG00000237978	KCNMB2 antisense RNA 1	5.41	5.18E−51	7.41	5.02E−249
ZFPM2‐AS1	ENSG00000251003	ZFPM2 antisense RNA 1	5.33	2.22E−88	5.16	1.42E−69

Abbreviations: LUAD, lung adenocarcinoma; LUSC, lung squamous cell carcinoma; NAT, natural antisense transcript.

### 3.2 Expression patterns of FAM38A‐AS and FAM38A mRNA in NSCLC

**Figure 3:**
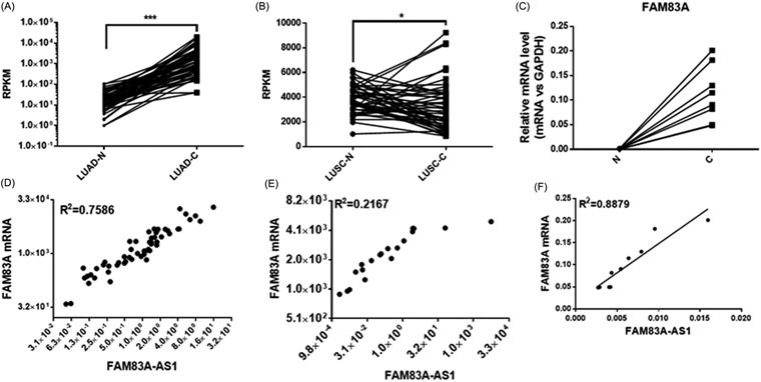
The correlation between FAM83A‐AS1 and FAM83A mRNA in LUAD and LUSC. (A,B) The correlation between FAM83A‐AS1 and FAM83A mRNA in LUAD (A), and LUSC (B) from the TCGA data. (C) The correlation between FAM83A‐AS1 and FAM83A mRNA in five LUAD tissues and five LUSC tissues as identified by qRT‐PCR. (D,E) The expression levels of FAM83A mRNA in normal and cancer tissues from LUAD (D) or LUSC (E) patients in the TCGA data. (F) The expression levels of FAM83A mRNA in paired normal and cancer tissues from five patients with LUAD and five with LUSC as revealed by qRT‐PCR. *P < 0.05; **P < 0.01; ***P < 0.001. LUAD, lung adenocarcinoma; LUSC, lung squamous cell carcinoma; mRNA; messenger RNA; qRT‐PCR, quantitative real‐time PCR; TGCA, The Cancer Genome Atlas

As a specific type of lncRNA, NATs are usually transcribed from the opposite DNA strand rather than the strand containing the sense transcripts of protein‐coding and non–protein‐coding genes and can partially overlap sense RNAs.^6^ An increasing number of studies has revealed that NATs usually regulate the expression of their cognate genes in a cis or trans manner.^7^ Therefore, we sought to reveal the role of FAM83A‐AS in the regulation of FAM83A. We delineated FAM83A‐AS and FAM83A messenger RNA (mRNA) expression patterns in LUAD and LUSC tissues from the TCGA data portal and NSCLC tissues collected from Nanjing Drum Tower Hospital. In the TCGA data, FAM83A mRNA was signiﬁcantly upregulated in LUAD and LUSC tissues (Figure 3A and B). In the matched normal control‐cancer tissue pairs obtained from 10 patients with NSCLC, FAM83A mRNA expression was also signiﬁcantly elevated (Figure 3C). More interestingly, we found that the expression of FAM83A‐AS positively correlated with FAM83A mRNA levels in LUAD and LUSC tissues from the TCGA data, as illustrated with Pearson's correlation scatter plots (Figure 3D and E). The results were confirmed in the matched normal control‐cancer tissue pairs obtained from 10 patients with NSCLC (Figure 3F). In summary, we speculate that FAM83A‐AS could regulate FAM83A in a cis manner.

### 3.3 FAM38A‐AS upregulates FAM38A at both the RNA and protein levels and promotes carcinogenesis

**Figure 4:**
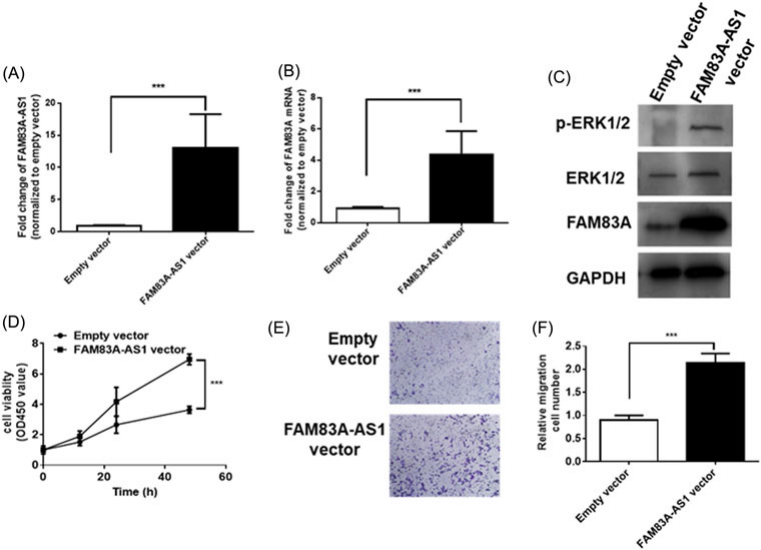
The function of FAM83A‐AS1 in lung cancer. (A,B) The relative levels of FAM83A‐AS1 (A) and FAM83A mRNA (B) in A549 cells transfected with an empty vector or the FAM83A‐AS1 overexpression vector. (C) Protein expression in A549 cells transfected with an empty vector or the FAM83A‐AS1 overexpression vector. (D) Growth curves of A549 cells transfected with an empty vector or the FAM83A‐AS1 overexpression vector. (E,F) Transwell assays of A549 cells transfected with an empty vector or the FAM83A‐AS1 overexpression vector. *P < 0.05; **P < 0.01; ***P < 0.001. mRNA, messenger RNA

The reciprocal relationship between FAM38A‐AS and FAM38A was further confirmed in the human alveolar adenocarcinoma cell line A549. We transiently overexpressed FAM38A‐AS in A549 cells using the FAM38A‐AS overexpression vector (Figure 4A). As anticipated, both the mRNA and protein levels of FAM38A were significantly upregulated in A549 cells (Figure 4B and C). FAM38A has been reported to enhance cancer cell proliferation and invasiveness by increasing the phosphorylation of ERK.^8^, ^9^, ^10^ Therefore, we detected ERK phosphorylation by Western blot analysis. As shown in Figure 4C, the phosphorylation of ERK was significantly increased because the FAM38A protein level was upregulated in the A549 cells transfected with the FAM38A‐AS overexpression vector. Then, we analysed the effect of FAM38A‐AS on cell proliferation and invasiveness. A CCK‐8 assay was utilized to analyse the proliferation of A549 cells transfected with an empty vector or the FAM38A‐AS vector. The results showed that FAM38A‐AS significantly increased the growth rate of cancer cells (Figure 4D). Transwell assays were performed to analyse the invasiveness of A549 cells. Similar to the results of the cell proliferation assay, FAM38A‐AS overexpression caused substantial increases in cancer cell migration (Figure 4E and 4F).

## 4 DISCUSSION

NATs are reverse‐complementary, at least in part, to the sequences of other endogenous sense transcripts and regulate the expression of their target genes.^2^ NATs, considered until recently to be transcriptional noise, are a very common phenomenon in human transcriptomes.^2^ Furthermore, they play indispensable functional roles in organ formation, cell differentiation, diseases and pathology. NATs have been shown to participate in gene regulation on nearly every level, including pretranscriptional, transcriptional and posttranscriptional, through DNA‐RNA, RNA‐RNA or protein‐RNA interactions. NSCLC is the leading cause of cancer‐related deaths worldwide.^1^ Recently, the profile of NATs in NSCLC has been reported and indicates that many NATs are dysregulated.^3^, ^11^, ^12^ In our present study, we analysed NATs in NSCLC by circlncRNAnet^4^ and identified 11 NATs that were significantly upregulated in both LUAD and LUSC in the TCGA data. These 11 NATs were further confirmed in patient with NSCLC tissues. FAM38A‐AS1, one of these 11 NATs, was markedly upregulated and highly expressed in cancer tissues.

There are two types of regulation between a NAT and its cognate sense mRNA, namely, discordant or concordant.^3^, ^13^ To explore the potential relationship between FAM38A‐AS1 and FAM38A, we used Pearson's correlation coefficient. The expression level of FAM83A‐AS was positively correlated with the FAM83A mRNA level in LUAD and LUSC samples from the TCGA data, which was similar to the results obtained in the matched normal control‐cancer tissue pairs obtained from 10 patients with NSCLC. We further confirmed the reciprocal relationship between FAM38A‐AS and FAM38A by overexpressing FAM38A‐AS in the human alveolar adenocarcinoma cell line A549. The results showed that both the mRNA and protein levels of FAM38A were significantly increased when FAM38A‐AS was overexpressed in A549 cells. These results showed that FAM83A‐AS could regulate FAM83A in a cis manner.

FAM38A is widely expressed in eukaryotic cells, especially in the epithelial cells of the skin, bladder, kidney, and lung. Many studies have found that deregulated FAM38A expression contributes to cancer in several tissue types, such as lung, breast and gastric tissues.^8^, ^9^, ^10^, ^14^, ^15^, ^16^, ^17^ Several studies have identified FAM83A as a candidate oncogene capable of enhancing cancer cell proliferation and invasiveness through the RAS/RAF/MEK/ERK and PI3K/AKT/mTOR pathways.^8^, ^9^, ^10^, ^14^, ^15^, ^16^, ^17^ In our study, we found that FAM83A‐AS could significantly increase the protein level of FAM38A and induce the phosphorylation of ERK, which results in the promotion of cancer cell proliferation and invasion.

In summary, our findings identified a novel AS, FAM38A‐AS, which could promote carcinogenesis by upregulating its cognate sense mRNA. FAM38A‐AS might serve as a new oncogene in the oncogenesis and progression of NSCLC and a promising prognostic and therapeutic target.

## CONFLICTS OF INTEREST

The authors declare that there are no conflicts of interest.

## AUTHOR CONTRIBUTIONS

TW designed the experiments. RS, ZJ, and AY performed the experiments and analyzed results. TW wrote the manuscript.

## Supporting information

Supporting Information
