# Supplementary material for: Long noncoding antisense RNA FAM83A‐AS1 promotes lung cancer cell progression by increasing FAM83A
Source: J Cell Biochem. 2019 Jan 18;120(6):10505–12. doi: 10.1002/jcb.28336 (PMC6590457; doi:10.1002/jcb.28336)
Supplement: Supplementary file 1 — Supporting Information [file JCB-120-10505-s001.doc]

**Long non-coding antisense RNA FAM83A-AS1 promotes lung cancer cell progression by increasing FAM83A**

**Rongxing Shi^1,*^, Zichen Jiao^1,2,*^, Ao Yu^1^, Tao Wang^1,2,#^**

**^1^**Department of cardiothoracic surgery, Nanjing Drum Tower Hospital, Nanjing University Medical School, Nanjing, China; **^2^**Department of cardiothoracic surgery, Nanjing Drum Tower Hospital, Nanjing Medical University, Nanjing, China

**Supplemental table**

**Table s1. The dysregulation ASs in LUAD.**

| gene | log2FoldChange | padj | Gene_full_name | Gene_old_names | Gene_other_names |
| --- | --- | --- | --- | --- | --- |
| DSCAM-AS1 | 7.170432 | 2.42E-25 | DSCAM antisense RNA 1 | NA | M41 |
| FOXD3-AS1 | 6.804552 | 1.26E-68 | FOXD3 antisense RNA 1 (head to head) | NA | pasFOXD3 |
| FAM83A-AS1 | 6.455432 | 1.09E-117 | FAM83A antisense RNA 1 | NA | HCCC11 |
| CLDN10-AS1 | 6.386443 | 4.23E-56 | CLDN10 antisense RNA 1 | NA | NA |
| AFAP1-AS1 | 6.268093 | 1.28E-126 | AFAP1 antisense RNA 1 | AFAP1AS;AFAP1-AS | MGC10981 |
| FEZF1-AS1 | 6.071111 | 9.92E-94 | FEZF1 antisense RNA 1 | NA | NA |
| BARX1-AS1 | 6.063958 | 7.93E-30 | BARX1 antisense RNA 1 (head to head) | NA | NA |
| NOVA1-AS1 | 5.66387 | 1.96E-27 | NOVA1 antisense RNA 1 (head to head) | C14orf22 | NA |
| POU6F2-AS2 | 5.537819 | 1.01E-19 | POU6F2 antisense RNA 2 | NA | FLJ12971 |
| NPSR1-AS1 | 5.501621 | 2.06E-41 | NPSR1 antisense RNA 1 | NA | AAA1;IMAGE:4827585 |
| BBOX1-AS1 | 5.484247 | 5.31E-61 | BBOX1 antisense RNA 1 | NA | NA |
| KCNMB2-AS1 | 5.412068 | 5.18E-51 | KCNMB2 antisense RNA 1 | NA | RP11-385J1.2 |
| ZFPM2-AS1 | 5.334155 | 2.22E-88 | ZFPM2 antisense RNA 1 | NA | NA |
| MNX1-AS1 | 5.151352 | 6.38E-85 | MNX1 antisense RNA 1 (head to head) | NA | NA |
| NAALADL2-AS2 | 4.800684 | 5.88E-24 | NAALADL2 antisense RNA 2 | NA | NA |
| DLX6-AS1 | 4.603899 | 4.38E-30 | DLX6 antisense RNA 1 | NCRNA00212 | FLJ34048;Evf-2 |
| SSTR5-AS1 | 3.940157 | 2.19E-14 | SSTR5 antisense RNA 1 | NA | NA |
| ELFN1-AS1 | 3.783284 | 1.13E-25 | ELFN1 antisense RNA 1 | NA | NA |
| MAFA-AS1 | 3.772992 | 3.24E-21 | MAFA antisense RNA 1 | NA | RP11-909N17.3;TCONS_00014882 |
| RUNDC3A-AS1 | 3.731965 | 1.29E-71 | RUNDC3A antisense RNA 1 | NA | NA |
| DSG1-AS1 | 3.556172 | 2.50E-07 | DSG1 antisense RNA 1 | NA | NA |
| PDX1-AS1 | 3.520393 | 4.53E-08 | PDX1 antisense RNA 1 | NA | NA |
| TRPM2-AS | 3.457133 | 7.84E-29 | TRPM2 antisense RNA | NA | TRPM2-AS1 |
| WASIR2 | 3.415159 | 2.19E-46 | WASH and IL9R antisense RNA 2 | NCRNA00286A | NA |
| STEAP2-AS1 | 3.378289 | 3.08E-20 | STEAP2 antisense RNA 1 | NA | NA |
| ZFHX4-AS1 | 3.376677 | 5.37E-10 | ZFHX4 antisense RNA 1 | NA | NA |
| ABCA9-AS1 | 3.369428 | 1.66E-13 | ABCA9 antisense RNA 1 | NA | NA |
| LSAMP-AS1 | 3.298191 | 1.44E-09 | LSAMP antisense RNA 1 | NA | NA |
| KIF25-AS1 | 3.231853 | 7.84E-16 | KIF25 antisense RNA 1 | C6orf54;NCRNA00300 | HGC6.1.1 |
| DPP10-AS1 | 3.200078 | 2.34E-20 | DPP10 antisense RNA 1 | NA | NA |
| CALML3-AS1 | 3.1739 | 1.59E-27 | CALML3 antisense RNA 1 | NA | NA |
| TFAP2A-AS1 | 3.064417 | 2.25E-49 | TFAP2A antisense RNA 1 | NA | NA |
| MNX1-AS2 | 3.025943 | 2.19E-28 | MNX1 antisense RNA 2 | NA | NA |
| C5orf66-AS1 | 3.025429 | 1.72E-09 | C5orf66 antisense RNA 1 | NA | CTC-276P9.1;Epist |
| POU6F2-AS1 | 2.970988 | 7.13E-08 | POU6F2 antisense RNA 1 | NA | NA |
| SATB2-AS1 | 2.904063 | 4.23E-20 | SATB2 antisense RNA 1 | NA | NA |
| NAALADL2-AS3 | 2.886204 | 0.000229 | NAALADL2 antisense RNA 3 | NA | NA |
| ARNTL2-AS1 | 2.870612 | 3.51E-11 | ARNTL2 antisense RNA 1 | NA | NA |
| VPS9D1-AS1 | 2.820106 | 9.81E-58 | VPS9D1 antisense RNA 1 | NA | NA |
| MTOR-AS1 | 2.810124 | 4.77E-06 | MTOR antisense RNA 1 | NA | NA |
| CHL1-AS1 | 2.772535 | 2.51E-12 | CHL1 antisense RNA 1 | NA | NA |
| CDKN2A-AS1 | 2.754635 | 2.46E-10 | CDKN2A antisense RNA 1 (head to head) | C9orf53 | bA149I2.3 |
| ST8SIA6-AS1 | 2.712518 | 4.27E-09 | ST8SIA6 antisense RNA 1 | NA | NA |
| ZBTB46-AS1 | 2.699484 | 5.71E-07 | ZBTB46 antisense RNA 1 | NA | NA |
| HNF1A-AS1 | 2.69402 | 6.10E-15 | HNF1A antisense RNA 1 | C12orf27;NCRNA00262 | FLJ38690 |
| PRKCA-AS1 | 2.690587 | 1.22E-07 | PRKCA antisense RNA 1 | NA | NA |
| VAC14-AS1 | 2.686066 | 4.23E-28 | VAC14 antisense RNA 1 | NA | NA |
| APCDD1L-AS1 | 2.664479 | 8.70E-18 | APCDD1L antisense RNA 1 (head to head) | NA | NA |
| RMDN2-AS1 | 2.657748 | 2.34E-28 | RMDN2 antisense RNA 1 | NA | NA |
| MKRN3-AS1 | 2.576778 | 3.27E-07 | MKRN3 antisense RNA 1 | ZNF127AS;MKRN3AS;MKRN3-AS | FNZ127;NCRNA00009;ZNF127-AS |
| CYP4A22-AS1 | 2.572119 | 4.39E-33 | CYP4A22 antisense RNA 1 | NA | ncRNA-a3 |
| C3orf67-AS1 | 2.5369 | 5.21E-05 | C3orf67 antisense RNA 1 | NA | NA |
| DPYD-AS1 | 2.534942 | 2.14E-12 | DPYD antisense RNA 1 | NA | NA |
| NHS-AS1 | 2.528042 | 2.60E-06 | NHS antisense RNA 1 | NA | NA |
| FAM222A-AS1 | 2.527733 | 1.84E-20 | FAM222A antisense RNA 1 | NA | NA |
| SAMSN1-AS1 | 2.485764 | 1.38E-10 | SAMSN1 antisense RNA 1 | NA | NA |
| PTGES2-AS1 | 2.483444 | 9.98E-22 | PTGES2 antisense RNA 1 (head to head) | NA | NA |
| VCAN-AS1 | 2.478633 | 4.98E-09 | VCAN antisense RNA 1 | NA | NA |
| DDX11-AS1 | 2.448727 | 4.06E-59 | DDX11 antisense RNA 1 | NA | NA |
| FGF12-AS2 | 2.448053 | 8.80E-08 | FGF12 antisense RNA 2 | NA | NA |
| FAM83C-AS1 | 2.418127 | 1.56E-15 | FAM83C antisense RNA 1 | C20orf120;NCRNA00154 | dJ614O4.3 |
| SPATA3-AS1 | 2.386192 | 9.86E-12 | SPATA3 antisense RNA 1 (head to head) | NA | NA |
| ARHGAP26-AS1 | 2.379864 | 3.54E-09 | ARHGAP26 antisense RNA 1 | NA | NA |
| SLC2A1-AS1 | 2.376074 | 9.48E-40 | SLC2A1 antisense RNA 1 | NA | NA |
| LEMD1-AS1 | 2.366156 | 1.19E-16 | LEMD1 antisense RNA 1 | NA | NA |
| RAPGEF4-AS1 | 2.360351 | 2.63E-06 | RAPGEF4 antisense RNA 1 | NA | NA |
| TRIM31-AS1 | 2.350897 | 1.62E-27 | TRIM31 antisense RNA 1 | NA | NA |
| PHACTR2-AS1 | 2.336627 | 5.74E-06 | PHACTR2 antisense RNA 1 | NA | NA |
| IL20RB-AS1 | 2.318951 | 1.86E-05 | IL20RB antisense RNA 1 | NA | NA |
| TCEAL3-AS1 | 2.314568 | 2.38E-07 | TCEAL3 antisense RNA 1 | NA | NA |
| ARHGEF3-AS1 | 2.288524 | 0.011039 | ARHGEF3 antisense RNA 1 | NA | NA |
| GRM5-AS1 | 2.263382 | 2.42E-08 | GRM5 antisense RNA 1 | NA | NA |
| SLC9A9-AS1 | 2.254719 | 0.020975 | SLC9A9 antisense RNA 1 | NA | NA |
| DEPDC1-AS1 | 2.235793 | 4.52E-11 | DEPDC1 antisense RNA 1 | NA | NA |
| PROX1-AS1 | 2.231618 | 1.07E-11 | PROX1 antisense RNA 1 | NA | NA |
| SNAP47-AS1 | 2.227063 | 5.82E-10 | SNAP47 antisense RNA 1 | SNAP47-IT1 | NA |
| FER1L6-AS2 | 2.222555 | 0.000132 | FER1L6 antisense RNA 2 | C8orf78 | FLJ32770 |
| FGF12-AS3 | 2.222185 | 1.32E-05 | FGF12 antisense RNA 3 | NA | NA |
| TBL1XR1-AS1 | 2.221296 | 3.39E-08 | TBL1XR1 antisense RNA 1 | NA | NA |
| ITGB5-AS1 | 2.205861 | 1.74E-06 | ITGB5 antisense RNA 1 | NA | NA |
| DOCK9-AS1 | 2.189664 | 0.005195 | DOCK9 antisense RNA 1 | NA | NA |
| SOX21-AS1 | 2.189591 | 1.89E-13 | SOX21 antisense RNA 1 (head to head) | NA | NA |
| KCNQ5-AS1 | 2.185921 | 0.006312 | KCNQ5 antisense RNA 1 | NA | NA |
| CLYBL-AS2 | 2.179047 | 2.35E-05 | CLYBL antisense RNA 2 | NA | NA |
| C9orf173-AS1 | 2.17482 | 1.94E-19 | C9orf173 antisense RNA 1 | NA | NA |
| TESC-AS1 | 2.153977 | 5.10E-07 | TESC antisense RNA 1 (head to head) | NA | NA |
| RNF144A-AS1 | 2.146618 | 8.68E-26 | RNF144A antisense RNA 1 | NA | NA |
| BFSP2-AS1 | 2.132093 | 5.37E-10 | BFSP2 antisense RNA 1 | NA | MGC2848 |
| CHODL-AS1 | 2.118885 | 0.000288 | CHODL antisense RNA 1 | C21orf39;NCRNA00157 | NA |
| USP12-AS1 | 2.086394 | 0.007209 | USP12 antisense RNA 1 | NA | NA |
| TTC3-AS1 | 2.080454 | 6.42E-14 | TTC3 antisense RNA 1 | NA | NA |
| LNX1-AS2 | 2.074016 | 5.94E-12 | LNX1 antisense RNA 2 | NA | NA |
| GPR1-AS | 2.071084 | 0.002563 | GPR1 antisense RNA | GPR1-AS1 | GPR1AS |
| AIRN | 2.051854 | 0.000769 | antisense of IGF2R non-protein coding RNA | NA | AIR;NCRNA00088;IGF2RAS;IGF2R-AS1 |
| STAU2-AS1 | 2.032825 | 3.45E-22 | STAU2 antisense RNA 1 | NA | NA |
| BIRC6-AS1 | 2.023146 | 0.00812 | BIRC6 antisense RNA 1 | NA | NA |
| THOC7-AS1 | 2.020466 | 2.80E-06 | THOC7 antisense RNA 1 | NA | NA |
| ZNF252P-AS1 | 2.020165 | 1.27E-28 | ZNF252P antisense RNA 1 | C8orf77 | NA |
| DLEU7-AS1 | 1.993126 | 1.26E-21 | DLEU7 antisense RNA 1 | NA | NA |
| PLS1-AS1 | 1.992074 | 0.015121 | PLS1 antisense RNA 1 | NA | NA |
| LNX1-AS1 | 1.983719 | 0.000358 | LNX1 antisense RNA 1 | NA | NA |
| C10orf71-AS1 | 1.973595 | 0.000185 | C10orf71 antisense RNA 1 | NA | NA |
| LARS2-AS1 | 1.960327 | 0.000162 | LARS2 antisense RNA 1 | NA | NA |
| DIO2-AS1 | 1.94192 | 0.003075 | DIO2 antisense RNA 1 | NA | NA |
| SYNPR-AS1 | 1.941059 | 1.08E-15 | SYNPR antisense RNA 1 | NA | NA |
| HS6ST2-AS1 | 1.937232 | 0.017077 | HS6ST2 antisense RNA 1 | NA | NA |
| LPP-AS1 | 1.937031 | 0.011603 | LPP antisense RNA 1 | NA | NA |
| UBE2Q1-AS1 | 1.929302 | 2.69E-23 | UBE2Q1 antisense RNA 1 | NA | NA |
| DARS-AS1 | 1.919185 | 3.00E-55 | DARS antisense RNA 1 | NA | NA |
| PRRX2-AS1 | 1.918337 | 9.08E-06 | PRRX2 antisense RNA 1 | NA | NA |
| SIRPG-AS1 | 1.914144 | 2.18E-09 | SIRPG antisense RNA 1 | NA | NA |
| OTX2-AS1 | 1.897152 | 0.00065 | OTX2 antisense RNA 1 (head to head) | NA | OTX2OS1 |
| MAGI1-AS1 | 1.890714 | 0.004153 | MAGI1 antisense RNA 1 | NA | NA |
| GDNF-AS1 | 1.886895 | 1.97E-09 | GDNF antisense RNA 1 (head to head) | NA | GDNFOS |
| EHMT2-AS1 | 1.885735 | 1.85E-10 | EHMT2 antisense RNA 1 | NA | NA |
| KCNMA1-AS3 | 1.878391 | 0.000187 | KCNMA1 antisense RNA 3 | NA | NA |
| TMLHE-AS1 | 1.876627 | 2.49E-09 | TMLHE antisense RNA 1 | NA | NA |
| PLCH1-AS2 | 1.855364 | 4.33E-06 | PLCH1 antisense RNA 2 | NA | NA |
| MACC1-AS1 | 1.851873 | 2.03E-05 | MACC1 antisense RNA 1 | NA | NA |
| BRWD1-AS1 | 1.851501 | 1.17E-07 | BRWD1 antisense RNA 1 | NA | NA |
| DPYD-AS2 | 1.850607 | 0.001619 | DPYD antisense RNA 2 | NA | NA |
| TXNDC12-AS1 | 1.837058 | 2.70E-06 | TXNDC12 antisense RNA 1 | NA | NA |
| WWC2-AS1 | 1.827691 | 0.002679 | WWC2 antisense RNA 1 | NA | NA |
| LATS2-AS1 | 1.821059 | 0.032749 | LATS2 antisense RNA 1 | NA | NA |
| MACROD2-AS1 | 1.820195 | 0.006685 | MACROD2 antisense RNA 1 | NCRNA00186 | BA318C17.1 |
| PAPPA-AS2 | 1.818702 | 0.002275 | PAPPA antisense RNA 2 | NA | AGU1 |
| TM4SF1-AS1 | 1.81465 | 2.91E-15 | TM4SF1 antisense RNA 1 | NA | NA |
| SH3PXD2A-AS1 | 1.811982 | 9.01E-11 | SH3PXD2A antisense RNA 1 | NA | NA |
| TMEM51-AS1 | 1.807011 | 9.97E-31 | TMEM51 antisense RNA 1 | C1orf126 | FLJ23703 |
| XXYLT1-AS1 | 1.805463 | 4.34E-06 | XXYLT1 antisense RNA 1 | NA | NA |
| COL18A1-AS2 | 1.795117 | 0.004928 | COL18A1 antisense RNA 2 | NA | NA |
| GPC6-AS1 | 1.776877 | 0.042028 | GPC6 antisense RNA 1 | NA | NA |
| PRRT3-AS1 | 1.774194 | 3.66E-27 | PRRT3 antisense RNA 1 | NA | NA |
| MRGPRG-AS1 | 1.773174 | 0.022245 | MRGPRG antisense RNA 1 | C11orf36 | FLJ36102;HSD-40 |
| SLC7A11-AS1 | 1.763389 | 3.52E-08 | SLC7A11 antisense RNA 1 | NA | NA |
| SLC26A4-AS1 | 1.761329 | 7.28E-08 | SLC26A4 antisense RNA 1 | NA | NA |
| ZNF571-AS1 | 1.759518 | 6.27E-15 | ZNF571 antisense RNA 1 | NA | NA |
| PTPRG-AS1 | 1.754573 | 4.48E-28 | PTPRG antisense RNA 1 | NA | NA |
| FMR1-AS1 | 1.753064 | 2.06E-07 | FMR1 antisense RNA 1 | FMR1AS;FMR1-AS | ASFMR1;FMR4 |
| KLHL7-AS1 | 1.752684 | 2.74E-21 | KLHL7 antisense RNA 1 (head to head) | NA | PLATAK |
| MYLK-AS2 | 1.751588 | 0.000433 | MYLK antisense RNA 2 | NA | NA |
| EVX1-AS | 1.74308 | 0.014044 | EVX1 antisense RNA | NA | EVX1-AS1 |
| UBXN10-AS1 | 1.739949 | 2.40E-17 | UBXN10 antisense RNA 1 | NA | NA |
| ATP6V1B1-AS1 | 1.734485 | 3.80E-06 | ATP6V1B1 antisense RNA 1 | NA | NA |
| IFNG-AS1 | 1.728085 | 5.34E-10 | IFNG antisense RNA 1 | NA | Tmevpg1;LincR-Ifng-3'AS;NEST |
| RAI1-AS1 | 1.72294 | 0.001016 | RAI1 antisense RNA 1 | NA | NA |
| HPN-AS1 | 1.721824 | 4.48E-17 | HPN antisense RNA 1 | NA | NA |
| NAV2-AS3 | 1.720039 | 5.22E-05 | NAV2 antisense RNA 3 | NA | NA |
| TRPC7-AS1 | 1.719705 | 0.001117 | TRPC7 antisense RNA 1 | NA | NA |
| ITPKB-AS1 | 1.718164 | 0.004162 | ITPKB antisense RNA 1 | NA | NA |
| PHKA1-AS1 | 1.717372 | 1.40E-05 | PHKA1 antisense RNA 1 | NA | NA |
| RDH10-AS1 | 1.714708 | 1.37E-12 | RDH10 antisense RNA 1 | NA | NA |
| LACTB2-AS1 | 1.707776 | 2.36E-18 | LACTB2 antisense RNA 1 | NA | NA |
| EDNRB-AS1 | 1.707289 | 2.14E-05 | EDNRB antisense RNA 1 | NA | NA |
| ITGB2-AS1 | 1.707031 | 4.17E-18 | ITGB2 antisense RNA 1 | NA | NA |
| UST-AS1 | 1.700886 | 0.020577 | UST antisense RNA 1 | NA | NA |
| ASTN2-AS1 | 1.70073 | 0.005104 | ASTN2 antisense RNA 1 | NA | NA |
| SPATA13-AS1 | 1.699664 | 0.001191 | SPATA13 antisense RNA 1 | NA | NA |
| PACERR | 1.697839 | 1.34E-12 | PTGS2 antisense NFKB1 complex-mediated expression regulator RNA | NA | PACER;PTGS2-AS1 |
| MPRIP-AS1 | 1.681561 | 7.14E-06 | MPRIP antisense RNA 1 | NA | NA |
| SLC8A1-AS1 | 1.678359 | 2.33E-08 | SLC8A1 antisense RNA 1 | NA | NA |
| CARS-AS1 | 1.666702 | 9.98E-07 | CARS antisense RNA 1 | NA | NA |
| MYCBP2-AS2 | 1.66532 | 0.000948 | MYCBP2 antisense RNA 2 | NA | NA |
| ZNF385D-AS1 | 1.661997 | 0.039716 | ZNF385D antisense RNA 1 | NA | NA |
| FOXD2-AS1 | 1.650815 | 1.54E-42 | FOXD2 antisense RNA 1 (head to head) | NA | MGC12982 |
| TLR8-AS1 | 1.650644 | 9.90E-06 | TLR8 antisense RNA 1 | NA | NA |
| PIK3CD-AS2 | 1.648944 | 1.45E-20 | PIK3CD antisense RNA 2 | NA | NA |
| UPK1A-AS1 | 1.648453 | 5.88E-05 | UPK1A antisense RNA 1 | NA | NA |
| ZMYM4-AS1 | 1.644947 | 1.16E-05 | ZMYM4 antisense RNA 1 | NA | NA |
| DENND5B-AS1 | 1.644086 | 6.29E-09 | DENND5B antisense RNA 1 | NA | NA |
| PRC1-AS1 | 1.634866 | 6.63E-15 | PRC1 antisense RNA 1 | NA | NA |
| EPHA5-AS1 | 1.622729 | 0.008844 | EPHA5 antisense RNA 1 | NA | NA |
| EXTL3-AS1 | 1.614418 | 1.16E-16 | EXTL3 antisense RNA 1 | C8orf50 | NA |
| ATP2A1-AS1 | 1.612523 | 2.68E-19 | ATP2A1 antisense RNA 1 | NA | NA |
| SHANK2-AS3 | 1.610892 | 0.006624 | SHANK2 antisense RNA 3 | C11orf76 | NA |
| MFI2-AS1 | 1.602081 | 2.70E-25 | MFI2 antisense RNA 1 | NA | NA |
| ANO1-AS2 | 1.586312 | 0.000612 | ANO1 antisense RNA 2 (head to head) | C11orf78;NCRNA00224 | NA |
| GPC6-AS2 | 1.581748 | 0.009195 | GPC6 antisense RNA 2 | NA | NA |
| OPA1-AS1 | 1.579956 | 2.75E-05 | OPA1 antisense RNA 1 | NA | NA |
| SIDT1-AS1 | 1.577978 | 8.43E-05 | SIDT1 antisense RNA 1 | NA | NA |
| CRYM-AS1 | 1.572133 | 3.01E-15 | CRYM antisense RNA 1 | NCRNA00169 | FLJ41766 |
| PHEX-AS1 | 1.57064 | 0.00148 | PHEX antisense RNA 1 | NA | NA |
| TMPO-AS1 | 1.564515 | 3.25E-31 | TMPO antisense RNA 1 | NA | NA |
| SRGAP2-AS1 | 1.563694 | 0.007063 | SRGAP2 antisense RNA 1 | NA | NA |
| PCSK6-AS1 | 1.559674 | 0.006437 | PCSK6 antisense RNA 1 | NA | NA |
| SMAD1-AS2 | 1.555349 | 0.009486 | SMAD1 antisense RNA 2 | NA | NA |
| RBM12B-AS1 | 1.554193 | 1.00E-19 | RBM12B antisense RNA 1 | C8orf39 | PRO1905 |
| CHL1-AS2 | 1.550785 | 1.06E-06 | CHL1 antisense RNA 2 | NA | NA |
| ATP13A5-AS1 | 1.545754 | 0.000787 | ATP13A5 antisense RNA 1 | NA | NA |
| GRM7-AS3 | 1.543111 | 0.032138 | GRM7 antisense RNA 3 | NA | NA |
| TMEM92-AS1 | 1.542921 | 9.03E-19 | TMEM92 antisense RNA 1 | NA | RP11-893F2.9;lncRNA-508851;TCONS_00025237 |
| EGLN3-AS1 | 1.533968 | 0.013066 | EGLN3 antisense RNA 1 | NA | NA |
| KIZ-AS1 | 1.531512 | 4.76E-09 | KIZ antisense RNA 1 | NA | NA |
| OSBPL10-AS1 | 1.52555 | 0.000187 | OSBPL10 antisense RNA 1 | NA | NA |
| SLCO4A1-AS1 | 1.51634 | 6.69E-07 | SLCO4A1 antisense RNA 1 | NA | NA |
| DNAH17-AS1 | 1.516116 | 2.64E-10 | DNAH17 antisense RNA 1 | NA | NA |
| ZNF630-AS1 | 1.515197 | 0.000287 | ZNF630 antisense RNA 1 | NA | NA |
| EIF1AX-AS1 | 1.513674 | 0.000464 | EIF1AX antisense RNA 1 | NA | NA |
| RHPN1-AS1 | 1.51203 | 7.39E-27 | RHPN1 antisense RNA 1 (head to head) | C8orf51 | MGC3113 |
| PLCB2-AS1 | 1.505378 | 1.41E-05 | PLCB2 antisense RNA 1 | NA | NA |
| FSIP2-AS1 | 1.503069 | 1.75E-07 | FSIP2 antisense RNA 1 | NA | NA |
| UCHL1-AS1 | 1.501338 | 0.000606 | UCHL1 antisense RNA 1 (head to head) | NA | NA |
| CSE1L-AS1 | 1.500917 | 1.62E-06 | CSE1L antisense RNA 1 | NA | NA |
| HIF1A-AS2 | 1.497614 | 1.30E-10 | HIF1A antisense RNA 2 | NA | 3'aHIF-1A;aHIF |
| WASF3-AS1 | 1.493143 | 0.038964 | WASF3 antisense RNA 1 | NA | NA |
| NFIA-AS1 | 1.489697 | 0.000498 | NFIA antisense RNA 1 | NA | RP5-833A20.1 |
| DLG3-AS1 | 1.487131 | 3.01E-13 | DLG3 antisense RNA 1 | NA | NA |
| C5orf66-AS2 | 1.483907 | 0.019167 | C5orf66 antisense RNA 2 | NA | NA |
| HDAC11-AS1 | 1.483729 | 5.99E-07 | HDAC11 antisense RNA 1 | NA | NA |
| XIAP-AS1 | 1.464578 | 0.00033 | XIAP antisense RNA 1 | NA | NA |
| ZBTB20-AS1 | 1.449701 | 1.85E-07 | ZBTB20 antisense RNA 1 | NA | NA |
| STPG2-AS1 | 1.446861 | 0.009414 | STPG2 antisense RNA 1 | NA | NA |
| SRD5A3-AS1 | 1.406572 | 5.88E-17 | SRD5A3 antisense RNA 1 | NA | NA |
| PKIA-AS1 | 1.406474 | 2.84E-05 | PKIA antisense RNA 1 | NA | NA |
| PLCXD2-AS1 | 1.404795 | 0.00148 | PLCXD2 antisense RNA 1 | NA | NA |
| POT1-AS1 | 1.39418 | 4.36E-14 | POT1 antisense RNA 1 | NA | NA |
| LGALS8-AS1 | 1.393777 | 1.01E-13 | LGALS8 antisense RNA 1 | NA | NA |
| CNOT10-AS1 | 1.391587 | 0.018752 | CNOT10 antisense RNA 1 | NA | NA |
| CASK-AS1 | 1.391574 | 1.70E-07 | CASK antisense RNA 1 | NA | NA |
| FUT8-AS1 | 1.385187 | 2.28E-19 | FUT8 antisense RNA 1 | NA | NA |
| RNF139-AS1 | 1.385041 | 4.81E-21 | RNF139 antisense RNA 1 (head to head) | NA | NA |
| ITCH-AS1 | 1.382555 | 0.01581 | ITCH antisense RNA 1 | NA | NA |
| ZNF793-AS1 | 1.381847 | 2.15E-13 | ZNF793 antisense RNA 1 (head to head) | NA | NA |
| GTF3C2-AS1 | 1.371439 | 3.97E-14 | GTF3C2 antisense RNA 1 | NA | NA |
| ENOX1-AS1 | 1.369183 | 0.010915 | ENOX1 antisense RNA 1 | NA | NA |
| NCOA7-AS1 | 1.358449 | 0.045575 | NCOA7 antisense RNA 1 | NA | NA |
| MAGEA8-AS1 | 1.35384 | 0.004182 | MAGEA8 antisense RNA 1 (head to head) | NA | RP5-869M20.2 |
| CYYR1-AS1 | 1.349863 | 0.002864 | cysteine/tyrosine-rich 1 antisense RNA 1 | NA | NA |
| DLX2-AS1 | 1.349387 | 0.023347 | DLX2 antisense RNA 1 (head to head) | NA | TCONS_00003049 |
| KIAA0196-AS1 | 1.339332 | 4.68E-09 | KIAA0196 antisense RNA 1 | NA | NA |
| UBE2R2-AS1 | 1.33479 | 1.24E-06 | UBE2R2 antisense RNA 1 | NA | NA |
| PTCHD1-AS | 1.32376 | 0.003437 | PTCHD1 antisense RNA (head to head) | NA | PTCHD1AS1;PTCHD1AS2;DDX53-AS1 |
| LMLN-AS1 | 1.317327 | 0.000454 | LMLN antisense RNA 1 | NA | NA |
| KIAA1614-AS1 | 1.316751 | 8.78E-08 | KIAA1614 antisense RNA 1 | NA | RP11-46A10.4 |
| B4GALT4-AS1 | 1.31248 | 0.006878 | B4GALT4 antisense RNA 1 | NA | NA |
| BIRC6-AS2 | 1.311881 | 1.15E-05 | BIRC6 antisense RNA 2 | NA | megamind |
| ST3GAL4-AS1 | 1.310112 | 2.25E-18 | ST3GAL4 antisense RNA 1 (head to head) | NA | DCPS-AS1;FLJ39051 |
| MIS18A-AS1 | 1.307436 | 6.44E-12 | MIS18A antisense RNA 1 | NA | NA |
| SBF2-AS1 | 1.299157 | 3.06E-36 | SBF2 antisense RNA 1 | NA | NA |
| JMJD1C-AS1 | 1.299111 | 2.05E-11 | JMJD1C antisense RNA 1 | NA | NA |
| CFAP44-AS1 | 1.298386 | 0.000366 | CFAP44 antisense RNA 1 | WDR52-AS1 | NA |
| SAMD12-AS1 | 1.297419 | 2.88E-17 | SAMD12 antisense RNA 1 | C8orf26;NCRNA00252 | NA |
| LOXL1-AS1 | 1.292355 | 6.59E-24 | LOXL1 antisense RNA 1 | NA | NA |
| STAM-AS1 | 1.290592 | 4.94E-14 | STAM antisense RNA 1 (head to head) | NA | locus3182 |
| PARD3-AS1 | 1.289309 | 2.30E-08 | PARD3 antisense RNA 1 | NA | NA |
| PTPRJ-AS1 | 1.287419 | 0.007471 | PTPRJ antisense RNA 1 | NA | NA |
| TTC21B-AS1 | 1.285542 | 0.000568 | TTC21B antisense RNA 1 | NA | NA |
| CAPN10-AS1 | 1.280269 | 1.47E-25 | CAPN10 antisense RNA 1 (head to head) | NA | locus959 |
| DDX39B-AS1 | 1.276914 | 7.61E-07 | DDX39B antisense RNA 1 | NA | NA |
| IGF2-AS | 1.276073 | 0.001896 | IGF2 antisense RNA | IGF2AS | PEG8;IGF2-AS1 |
| JARID2-AS1 | 1.275113 | 8.53E-07 | JARID2 antisense RNA 1 | NA | NA |
| JRKL-AS1 | 1.271782 | 0.019464 | JRKL antisense RNA 1 | NA | NA |
| CELSR3-AS1 | 1.269062 | 2.31E-14 | CELSR3 antisense RNA 1 (head to head) | NA | NA |
| SLC25A25-AS1 | 1.268628 | 4.13E-15 | SLC25A25 antisense RNA 1 | NA | NA |
| LEF1-AS1 | 1.262484 | 3.99E-13 | LEF1 antisense RNA 1 | NA | NA |
| CLSTN2-AS1 | 1.262437 | 0.012364 | CLSTN2 antisense RNA 1 | NA | NA |
| ROR1-AS1 | 1.26095 | 6.29E-07 | ROR1 antisense RNA 1 | NA | NA |
| PRMT5-AS1 | 1.254066 | 2.01E-15 | PRMT5 antisense RNA 1 | NA | NA |
| MCM8-AS1 | 1.250014 | 0.00035 | MCM8 antisense RNA 1 | NA | NA |
| ASMTL-AS1 | 1.248682 | 1.05E-09 | ASMTL antisense RNA 1 | CXYorf2;NCRNA00105;ASMTLAS;ASMTL-AS | FLJ13330 |
| STEAP3-AS1 | 1.247138 | 1.72E-10 | STEAP3 antisense RNA 1 | NA | NA |
| COL18A1-AS1 | 1.246144 | 0.004141 | COL18A1 antisense RNA 1 | C21orf123;NCRNA00175 | PRED80 |
| SATB1-AS1 | 1.243852 | 9.29E-11 | SATB1 antisense RNA 1 | NA | NA |
| CACNA1G-AS1 | 1.242564 | 4.66E-06 | CACNA1G antisense RNA 1 | NA | NA |
| KLHL6-AS1 | 1.241078 | 0.008553 | KLHL6 antisense RNA 1 | NA | NA |
| RALY-AS1 | 1.240721 | 6.56E-30 | RALY antisense RNA 1 | NA | NA |
| SZT2-AS1 | 1.240159 | 1.15E-05 | SZT2 antisense RNA 1 | NA | NA |
| WT1-AS | 1.234275 | 0.000175 | WT1 antisense RNA | WIT1 | WIT-1;WT1AS;WT1-AS1 |
| SMAD5-AS1 | 1.233691 | 4.36E-05 | SMAD5 antisense RNA 1 | SMAD5OS | DAMS |
| MYHAS | 1.229749 | 3.67E-05 | myosin heavy chain gene cluster antisense RNA | NA | NA |
| DIAPH2-AS1 | 1.221696 | 5.57E-06 | DIAPH2 antisense RNA 1 | NA | NA |
| C9orf41-AS1 | 1.213641 | 2.40E-05 | C9orf41 antisense RNA 1 | NA | NA |
| HCFC1-AS1 | 1.206779 | 1.48E-05 | HCFC1 antisense RNA 1 | NA | NA |
| SPATA17-AS1 | 1.201562 | 0.012739 | SPATA17 antisense RNA 1 | NA | NA |
| TET2-AS1 | 1.198493 | 0.002846 | TET2 antisense RNA 1 | NA | NA |
| HMMR-AS1 | 1.196095 | 0.001295 | HMMR antisense RNA 1 | NA | NA |
| MAFG-AS1 | 1.195876 | 1.22E-21 | MAFG antisense RNA 1 (head to head) | NA | NA |
| OVOL1-AS1 | 1.194042 | 0.000149 | OVOL1 antisense RNA 1 | NA | NA |
| PRKG1-AS1 | 1.194011 | 9.99E-05 | PRKG1 antisense RNA 1 | NA | NA |
| FARSA-AS1 | 1.193352 | 0.000604 | FARSA antisense RNA 1 | NA | NA |
| CSTF3-AS1 | 1.192777 | 2.65E-07 | CSTF3 antisense RNA 1 (head to head) | NA | NA |
| ADARB2-AS1 | 1.191134 | 0.047612 | ADARB2 antisense RNA 1 | C10orf109;NCRNA00168 | bA466B20.1 |
| UFL1-AS1 | 1.188776 | 0.000361 | UFL1 antisense RNA 1 | NA | NA |
| SIX3-AS1 | 1.187486 | 0.019338 | SIX3 antisense RNA 1 | NA | NA |
| KCNMA1-AS1 | 1.181625 | 0.00032 | KCNMA1 antisense RNA 1 | NA | NA |
| TBX18-AS1 | 1.179807 | 0.032253 | TBX18 antisense RNA 1 | NA | NA |
| CERS6-AS1 | 1.178478 | 1.04E-05 | CERS6 antisense RNA 1 | NA | NA |
| KCNQ1OT1 | 1.175064 | 2.49E-10 | KCNQ1 opposite strand/antisense transcript 1 (non-protein coding) | NA | KvDMR1;KCNQ1-AS2;KvLQT1-AS;LIT1;NCRNA00012 |
| RFPL1S | 1.174991 | 3.77E-06 | RFPL1 antisense RNA 1 | RFPL1-AS1 | RFPL1-AS;NCRNA00006 |
| PRR7-AS1 | 1.171139 | 1.91E-09 | PRR7 antisense RNA 1 | NA | NA |
| HORMAD2-AS1 | 1.157365 | 0.000216 | HORMAD2 antisense RNA 1 | NA | MTMR3-AS1;NONHSAG033653 |
| KDM4A-AS1 | 1.151368 | 4.86E-18 | KDM4A antisense RNA 1 | NA | NA |
| FOCAD-AS1 | 1.145198 | 4.88E-05 | FOCAD antisense RNA 1 | NA | NA |
| IGF2BP2-AS1 | 1.142444 | 0.005605 | IGF2BP2 antisense RNA 1 | C3orf65 | FLJ32900 |
| SRRM2-AS1 | 1.136207 | 2.26E-15 | SRRM2 antisense RNA 1 | NA | NA |
| BEAN1-AS1 | 1.13612 | 2.33E-06 | BEAN1 antisense RNA 1 | NA | NA |
| PAQR9-AS1 | 1.132251 | 0.036308 | PAQR9 antisense RNA 1 | NA | NA |
| DLGAP1-AS5 | 1.132013 | 0.009572 | DLGAP1 antisense RNA 5 | NA | NA |
| MYB-AS1 | 1.12404 | 0.030039 | MYB antisense RNA 1 | MYBAS;MYB-AS | RP1-32B1.3;NCRNA00209 |
| LINGO1-AS1 | 1.118878 | 0.018534 | LINGO1 antisense RNA 1 | NA | NA |
| COL5A1-AS1 | 1.116618 | 0.029112 | COL5A1 antisense RNA 1 | C9orf104 | bA54A22.4 |
| FOXP4-AS1 | 1.114754 | 7.87E-09 | FOXP4 antisense RNA 1 | NA | NA |
| FRMD6-AS1 | 1.103212 | 7.42E-09 | FRMD6 antisense RNA 1 | C14orf82 | NA |
| NFYC-AS1 | 1.09307 | 4.09E-14 | NFYC antisense RNA 1 | NA | 0808y08y |
| PLCE1-AS1 | 1.086917 | 0.001132 | PLCE1 antisense RNA 1 | NA | NA |
| AP4B1-AS1 | 1.076128 | 1.80E-10 | AP4B1 antisense RNA 1 | NA | NA |
| DSCAS | 1.073805 | 1.58E-05 | DSC1/DSC2 antisense RNA | NA | NA |
| LAMP5-AS1 | 1.072968 | 0.004933 | LAMP5 antisense RNA 1 | NA | NA |
| CRTC3-AS1 | 1.065989 | 7.55E-11 | CRTC3 antisense RNA 1 | NA | NA |
| ATP11A-AS1 | 1.061693 | 0.008791 | ATP11A antisense RNA 1 | NA | NA |
| MAP3K14-AS1 | 1.058769 | 4.57E-23 | MAP3K14 antisense RNA 1 | NA | NA |
| FBXL19-AS1 | 1.044451 | 4.29E-14 | FBXL19 antisense RNA 1 (head to head) | NCRNA00095 | MGC125469;MGC125470;MGC125472 |
| CHKB-AS1 | 1.042292 | 6.30E-14 | CHKB antisense RNA 1 (head to head) | NA | NA |
| TMEM147-AS1 | 1.04166 | 2.48E-17 | TMEM147 antisense RNA 1 | NA | NA |
| RRM1-AS1 | 1.03851 | 0.002477 | RRM1 antisense RNA 1 | NA | NA |
| TAF1A-AS1 | 1.035218 | 3.09E-16 | TAF1A antisense RNA 1 | NA | NA |
| SCAANT1 | 1.031875 | 3.35E-06 | SCA7/ATXN7 antisense RNA 1 | NA | ATXN7-AS1 |
| ACTN1-AS1 | 1.029145 | 1.36E-05 | ACTN1 antisense RNA 1 | C14orf84 | NA |
| DLEU1-AS1 | 1.02814 | 0.041647 | DLEU1 antisense RNA 1 | NA | LINC01308 |
| DLGAP1-AS3 | 1.012962 | 0.03354 | DLGAP1 antisense RNA 3 | NA | NA |
| PCCA-AS1 | 1.008073 | 0.006162 | PCCA antisense RNA 1 | NA | NA |
| NCBP2-AS1 | 1.002161 | 4.69E-08 | NCBP2 antisense RNA 1 | NA | NA |
| CCDC13-AS1 | -1.00195 | 4.17E-09 | CCDC13 antisense RNA 1 | NA | NA |
| TTLL10-AS1 | -1.00867 | 0.000656 | TTLL10 antisense RNA 1 | NA | NA |
| TMEM5-AS1 | -1.02877 | 6.91E-06 | TMEM5 antisense RNA 1 | NA | NA |
| CCND2-AS2 | -1.03494 | 7.66E-05 | CCND2 antisense RNA 2 | NA | NA |
| LMF1-AS1 | -1.0385 | 3.43E-06 | LMF1 antisense RNA 1 | NA | NA |
| SLC14A2-AS1 | -1.04252 | 0.000134 | SLC14A2 antisense RNA 1 | NA | NA |
| CNTN4-AS1 | -1.05052 | 0.006487 | CNTN4 antisense RNA 1 | NA | NA |
| RBPMS-AS1 | -1.05556 | 1.34E-08 | RBPMS antisense RNA 1 | NA | NA |
| CYP17A1-AS1 | -1.06234 | 8.94E-05 | CYP17A1 antisense RNA 1 | CYP17A1OS | bA753C18.3 |
| CERS3-AS1 | -1.06383 | 0.004422 | CERS3 antisense RNA 1 | NA | NA |
| SMARCA5-AS1 | -1.06724 | 5.88E-05 | SMARCA5 antisense RNA 1 | NA | NA |
| ADIRF-AS1 | -1.09997 | 2.90E-10 | ADIRF antisense RNA 1 | NA | NA |
| LBX1-AS1 | -1.10135 | 9.52E-07 | LBX1 antisense RNA 1 (head to head) | NA | FLJ41350 |
| CA3-AS1 | -1.10154 | 8.44E-09 | CA3 antisense RNA 1 | NA | NA |
| WDR11-AS1 | -1.12961 | 2.79E-06 | WDR11 antisense RNA 1 | NA | NA |
| ARHGEF7-AS2 | -1.13289 | 0.000496 | ARHGEF7 antisense RNA 2 | NA | NA |
| ATP13A4-AS1 | -1.13541 | 0.002481 | ATP13A4 antisense RNA 1 | NA | NA |
| SEMA3B-AS1 | -1.15599 | 2.44E-12 | SEMA3B antisense RNA 1 (head to head) | NA | NA |
| DPH6-AS1 | -1.17715 | 1.37E-12 | DPH6 antisense RNA 1 (head to head) | ATPBD4-AS1 | NA |
| GAS6-AS2 | -1.1854 | 1.62E-13 | GAS6 antisense RNA 2 (head to head) | NA | FLJ44054 |
| VIPR1-AS1 | -1.19383 | 1.68E-07 | VIPR1 antisense RNA 1 | NA | NA |
| APOA1-AS | -1.19667 | 2.22E-08 | APOA1 antisense RNA | NA | NA |
| PDZRN3-AS1 | -1.20223 | 0.016492 | PDZRN3 antisense RNA 1 | NA | NA |
| SMC2-AS1 | -1.22667 | 1.04E-08 | SMC2 antisense RNA 1 (head to head) | NA | NA |
| EPB41L4A-AS2 | -1.24748 | 2.32E-25 | EPB41L4A antisense RNA 2 (head to head) | NA | FLJ11235 |
| JAZF1-AS1 | -1.25025 | 2.68E-11 | JAZF1 antisense RNA 1 | NA | NA |
| FRMD6-AS2 | -1.25934 | 0.013203 | FRMD6 antisense RNA 2 | NA | NA |
| AQP4-AS1 | -1.26154 | 3.93E-06 | AQP4 antisense RNA 1 | C18orf16;CHST9-AS1 | FLJ30507 |
| CPEB1-AS1 | -1.26191 | 3.23E-06 | CPEB1 antisense RNA 1 | NA | NA |
| SH3RF3-AS1 | -1.28199 | 2.21E-20 | SH3RF3 antisense RNA 1 | NA | NA |
| USP30-AS1 | -1.28774 | 1.06E-16 | USP30 antisense RNA 1 | NA | NA |
| TARID | -1.33379 | 3.51E-07 | TCF21 antisense RNA inducing promoter demethylation | EYA4-AS1 | NA |
| TRHDE-AS1 | -1.33971 | 0.000132 | TRHDE antisense RNA 1 | NA | NA |
| P4HA2-AS1 | -1.36598 | 4.82E-10 | P4HA2 antisense RNA 1 | NA | NA |
| COL4A2-AS1 | -1.3856 | 3.88E-11 | COL4A2 antisense RNA 1 | NA | NA |
| ADAMTS9-AS2 | -1.41059 | 5.05E-11 | ADAMTS9 antisense RNA 2 | NA | NA |
| C8orf34-AS1 | -1.41089 | 1.45E-07 | C8orf34 antisense RNA 1 | NA | NA |
| PTENP1-AS | -1.41893 | 4.47E-06 | PTENP1 antisense RNA | NA | PTENpg1-asRNA |
| GFOD1-AS1 | -1.42634 | 0.000134 | GFOD1 antisense RNA 1 | NA | NA |
| MRGPRF-AS1 | -1.43189 | 1.31E-11 | MRGPRF antisense RNA 1 | NA | NA |
| GATA6-AS1 | -1.51257 | 1.92E-09 | GATA6 antisense RNA 1 (head to head) | NA | locus5689 |
| MEOX2-AS1 | -1.51286 | 1.44E-12 | MEOX2 antisense RNA 1 (head to head) | NA | NA |
| FGF14-AS2 | -1.56549 | 2.39E-22 | FGF14 antisense RNA 2 | NA | NA |
| MAGI2-AS3 | -1.57396 | 6.80E-31 | MAGI2 antisense RNA 3 | NA | NA |
| RHOXF1-AS1 | -1.58487 | 3.91E-10 | RHOXF1 antisense RNA 1 | NA | NA |
| HHIP-AS1 | -1.58528 | 1.03E-10 | HHIP antisense RNA 1 | NA | NA |
| PGM5P3-AS1 | -1.59232 | 7.92E-10 | PGM5P3 antisense RNA 1 | NA | FAM233B |
| WWC2-AS2 | -1.60228 | 3.03E-32 | WWC2 antisense RNA 2 | C4orf38 | FLJ30277 |
| P3H2-AS1 | -1.6139 | 1.05E-07 | P3H2 antisense RNA 1 | LEPREL1-AS1 | NA |
| VWA8-AS1 | -1.6186 | 5.40E-12 | VWA8 antisense RNA 1 (head to head) | NA | NA |
| FAM167A-AS1 | -1.63263 | 3.60E-06 | FAM167A antisense RNA 1 | C8orf12 | NA |
| LHFPL3-AS1 | -1.66184 | 3.15E-06 | LHFPL3 antisense RNA 1 | NA | NA |
| AGBL1-AS1 | -1.72295 | 0.000985 | AGBL1 antisense RNA 1 | NA | NA |
| RAMP2-AS1 | -1.72683 | 7.49E-17 | RAMP2 antisense RNA 1 | NA | NA |
| EP300-AS1 | -1.74999 | 2.67E-32 | EP300 antisense RNA 1 | NA | NA |
| TBX5-AS1 | -1.78866 | 1.01E-34 | TBX5 antisense RNA 1 | NA | NA |
| FAM181A-AS1 | -1.81769 | 4.45E-07 | FAM181A antisense RNA 1 | C14orf86 | NA |
| MBNL1-AS1 | -1.82074 | 1.42E-33 | MBNL1 antisense RNA 1 | NA | NA |
| GPC5-AS1 | -1.82702 | 0.000313 | GPC5 antisense RNA 1 | NA | NA |
| HHATL-AS1 | -1.88633 | 1.49E-07 | HHATL antisense RNA 1 | NA | NA |
| UMODL1-AS1 | -1.93563 | 2.01E-11 | UMODL1 antisense RNA 1 | C21orf128 | FLJ33471 |
| NALCN-AS1 | -1.93878 | 3.30E-12 | NALCN antisense RNA 1 | NA | NA |
| PKNOX2-AS1 | -1.961 | 8.38E-08 | PKNOX2 antisense RNA 1 (head to head) | NA | NA |
| PGM5P4-AS1 | -1.96383 | 2.64E-16 | PGM5P4 antisense RNA 1 | NA | FAM233C |
| PGM5-AS1 | -1.97618 | 3.19E-18 | PGM5 antisense RNA 1 | NA | FAM233A |
| TBX2-AS1 | -2.01868 | 9.27E-38 | TBX2 antisense RNA 1 | NA | NA |
| PACRG-AS1 | -2.02569 | 3.11E-12 | PACRG antisense RNA 1 | NA | NA |
| STARD13-AS | -2.03826 | 3.53E-18 | STARD13 antisense RNA | STARD13-AS2 | NA |
| CADM3-AS1 | -2.0495 | 1.74E-18 | CADM3 antisense RNA 1 | NA | CTA-134P22.2 |
| LHFPL3-AS2 | -2.05876 | 5.24E-12 | LHFPL3 antisense RNA 2 | NA | NA |
| ADAMTS9-AS1 | -2.14914 | 1.46E-18 | ADAMTS9 antisense RNA 1 | NA | NA |
| SRGAP3-AS2 | -2.16053 | 3.66E-08 | SRGAP3 antisense RNA 2 | NA | NA |
| F11-AS1 | -2.22092 | 7.86E-14 | F11 antisense RNA 1 | NA | NA |
| MED4-AS1 | -2.22421 | 3.53E-37 | MED4 antisense RNA 1 | MED4-AS | NA |
| PTPRD-AS1 | -2.26328 | 1.62E-36 | PTPRD antisense RNA 1 | NA | NA |
| HLX-AS1 | -2.33735 | 9.03E-33 | HLX antisense RNA 1 | NA | NA |
| MGAT3-AS1 | -2.37003 | 7.58E-16 | MGAT3 antisense RNA 1 | NA | TapSAKI |
| NAV2-AS2 | -2.38482 | 1.22E-16 | NAV2 antisense RNA 2 | NA | NA |
| MYO16-AS1 | -2.39993 | 1.87E-11 | MYO16 antisense RNA 1 | NA | NA |
| HID1-AS1 | -2.8024 | 4.23E-75 | HID1 antisense RNA 1 | NA | NA |
| FGF10-AS1 | -3.08026 | 1.65E-17 | FGF10 antisense RNA 1 | NA | RP11-473L15.2 |
| PACRG-AS3 | -3.34885 | 4.19E-17 | PACRG antisense RNA 3 | NA | NA |
| LANCL1-AS1 | -3.53721 | 9.18E-65 | LANCL1 antisense RNA 1 | NA | LANCLOT |

**Table s2. The dysregulation ASs in LU**SC.

| gene | log2FoldChange | padj | Gene_full_name | Gene_old_names | Gene_other_names |
| --- | --- | --- | --- | --- | --- |
| C5orf66-AS1 | 7.932739 | 3.99E-67 | C5orf66 antisense RNA 1 | NA | CTC-276P9.1;Epist |
| FOXD3-AS1 | 7.524751 | 9.98E-94 | FOXD3 antisense RNA 1 (head to head) | NA | pasFOXD3 |
| POU6F2-AS2 | 7.453979 | 1.72E-91 | POU6F2 antisense RNA 2 | NA | FLJ12971 |
| KCNMB2-AS1 | 7.414313 | 5.02E-249 | KCNMB2 antisense RNA 1 | NA | RP11-385J1.2 |
| DSG1-AS1 | 7.02825 | 3.97E-69 | DSG1 antisense RNA 1 | NA | NA |
| DLX6-AS1 | 6.959521 | 8.35E-140 | DLX6 antisense RNA 1 | NCRNA00212 | FLJ34048;Evf-2 |
| BBOX1-AS1 | 6.813446 | 1.82E-206 | BBOX1 antisense RNA 1 | NA | NA |
| NPSR1-AS1 | 6.68321 | 1.24E-64 | NPSR1 antisense RNA 1 | NA | AAA1;IMAGE:4827585 |
| BARX1-AS1 | 6.556188 | 4.28E-48 | BARX1 antisense RNA 1 (head to head) | NA | NA |
| FAM83A-AS1 | 6.304675 | 2.56E-73 | FAM83A antisense RNA 1 | NA | HCCC11 |
| CALML3-AS1 | 6.126472 | 2.85E-138 | CALML3 antisense RNA 1 | NA | NA |
| NAALADL2-AS2 | 6.049585 | 2.33E-47 | NAALADL2 antisense RNA 2 | NA | NA |
| FEZF1-AS1 | 5.905212 | 9.19E-85 | FEZF1 antisense RNA 1 | NA | NA |
| ZFHX4-AS1 | 5.521447 | 8.56E-27 | ZFHX4 antisense RNA 1 | NA | NA |
| NOVA1-AS1 | 5.347448 | 1.45E-24 | NOVA1 antisense RNA 1 (head to head) | C14orf22 | NA |
| ZFPM2-AS1 | 5.158531 | 1.42E-69 | ZFPM2 antisense RNA 1 | NA | NA |
| DLX2-AS1 | 5.08494 | 1.10E-32 | DLX2 antisense RNA 1 (head to head) | NA | TCONS_00003049 |
| MAFA-AS1 | 5.049187 | 4.76E-38 | MAFA antisense RNA 1 | NA | RP11-909N17.3;TCONS_00014882 |
| LSAMP-AS1 | 4.9608 | 4.68E-43 | LSAMP antisense RNA 1 | NA | NA |
| IL20RB-AS1 | 4.842679 | 2.23E-28 | IL20RB antisense RNA 1 | NA | NA |
| MNX1-AS1 | 4.810455 | 2.68E-41 | MNX1 antisense RNA 1 (head to head) | NA | NA |
| FER1L6-AS2 | 4.797181 | 8.37E-15 | FER1L6 antisense RNA 2 | C8orf78 | FLJ32770 |
| TRPM2-AS | 4.67117 | 1.50E-70 | TRPM2 antisense RNA | NA | TRPM2-AS1 |
| AFAP1-AS1 | 4.524053 | 2.72E-32 | AFAP1 antisense RNA 1 | AFAP1AS;AFAP1-AS | MGC10981 |
| ABCA9-AS1 | 4.523729 | 5.75E-45 | ABCA9 antisense RNA 1 | NA | NA |
| OTX2-AS1 | 4.450857 | 1.80E-19 | OTX2 antisense RNA 1 (head to head) | NA | OTX2OS1 |
| ARNTL2-AS1 | 4.254489 | 2.15E-29 | ARNTL2 antisense RNA 1 | NA | NA |
| SSTR5-AS1 | 4.178875 | 6.56E-18 | SSTR5 antisense RNA 1 | NA | NA |
| SOX21-AS1 | 4.080594 | 3.79E-65 | SOX21 antisense RNA 1 (head to head) | NA | NA |
| OSTM1-AS1 | 4.024047 | 1.97E-13 | OSTM1 antisense RNA 1 | NA | NA |
| MKRN3-AS1 | 3.979833 | 1.14E-20 | MKRN3 antisense RNA 1 | ZNF127AS;MKRN3AS;MKRN3-AS | FNZ127;NCRNA00009;ZNF127-AS |
| DEPDC1-AS1 | 3.787271 | 4.71E-32 | DEPDC1 antisense RNA 1 | NA | NA |
| SH3PXD2A-AS1 | 3.777333 | 4.64E-45 | SH3PXD2A antisense RNA 1 | NA | NA |
| GDNF-AS1 | 3.767495 | 4.60E-28 | GDNF antisense RNA 1 (head to head) | NA | GDNFOS |
| APCDD1L-AS1 | 3.737485 | 1.88E-29 | APCDD1L antisense RNA 1 (head to head) | NA | NA |
| GRM5-AS1 | 3.664123 | 8.02E-16 | GRM5 antisense RNA 1 | NA | NA |
| VPS9D1-AS1 | 3.648989 | 5.83E-105 | VPS9D1 antisense RNA 1 | NA | NA |
| ELFN1-AS1 | 3.635847 | 1.02E-24 | ELFN1 antisense RNA 1 | NA | NA |
| C15orf59-AS1 | 3.535634 | 1.52E-17 | C15orf59 antisense RNA 1 | NA | TCONS_00023459 |
| RAPGEF4-AS1 | 3.433714 | 3.03E-19 | RAPGEF4 antisense RNA 1 | NA | NA |
| SATB2-AS1 | 3.407431 | 2.63E-36 | SATB2 antisense RNA 1 | NA | NA |
| CDKN2A-AS1 | 3.392462 | 2.79E-16 | CDKN2A antisense RNA 1 (head to head) | C9orf53 | bA149I2.3 |
| SLC2A1-AS1 | 3.361639 | 1.15E-79 | SLC2A1 antisense RNA 1 | NA | NA |
| TFAP2A-AS1 | 3.334029 | 7.21E-89 | TFAP2A antisense RNA 1 | NA | NA |
| RUNDC3A-AS1 | 3.291784 | 4.07E-51 | RUNDC3A antisense RNA 1 | NA | NA |
| CHODL-AS1 | 3.279631 | 1.30E-13 | CHODL antisense RNA 1 | C21orf39;NCRNA00157 | NA |
| IGF2BP2-AS1 | 3.218858 | 5.40E-25 | IGF2BP2 antisense RNA 1 | C3orf65 | FLJ32900 |
| KIRREL3-AS1 | 3.180903 | 5.45E-09 | KIRREL3 antisense RNA 1 | NA | NA |
| ATP6V1B1-AS1 | 3.165519 | 9.30E-21 | ATP6V1B1 antisense RNA 1 | NA | NA |
| TCF4-AS1 | 3.140246 | 1.65E-16 | TCF4 antisense RNA 1 | NA | MIR4529HG |
| B3GALT5-AS1 | 3.119936 | 1.57E-15 | B3GALT5 antisense RNA 1 | C21orf88 | NA |
| ATP13A5-AS1 | 3.101721 | 8.37E-11 | ATP13A5 antisense RNA 1 | NA | NA |
| EGLN3-AS1 | 3.08458 | 4.02E-09 | EGLN3 antisense RNA 1 | NA | NA |
| PDX1-AS1 | 3.054562 | 1.10E-05 | PDX1 antisense RNA 1 | NA | NA |
| CYP4A22-AS1 | 3.038453 | 2.52E-46 | CYP4A22 antisense RNA 1 | NA | ncRNA-a3 |
| XXYLT1-AS1 | 3.037041 | 5.14E-21 | XXYLT1 antisense RNA 1 | NA | NA |
| MFI2-AS1 | 3.001645 | 7.25E-79 | MFI2 antisense RNA 1 | NA | NA |
| DIO2-AS1 | 2.967925 | 1.43E-06 | DIO2 antisense RNA 1 | NA | NA |
| STEAP2-AS1 | 2.958118 | 1.19E-20 | STEAP2 antisense RNA 1 | NA | NA |
| FGF12-AS3 | 2.955018 | 1.16E-10 | FGF12 antisense RNA 3 | NA | NA |
| DDX11-AS1 | 2.947328 | 1.93E-86 | DDX11 antisense RNA 1 | NA | NA |
| LHX5-AS1 | 2.94625 | 4.59E-08 | LHX5 antisense RNA 1 (head to head) | NA | locus4010 |
| FSIP2-AS1 | 2.935552 | 5.54E-30 | FSIP2 antisense RNA 1 | NA | NA |
| POU6F2-AS1 | 2.930299 | 1.31E-11 | POU6F2 antisense RNA 1 | NA | NA |
| TBX18-AS1 | 2.929145 | 8.01E-11 | TBX18 antisense RNA 1 | NA | NA |
| PAUPAR | 2.92037 | 3.67E-06 | PAX6 upstream antisense RNA | NA | NA |
| ELOVL2-AS1 | 2.907199 | 5.46E-10 | ELOVL2 antisense RNA 1 | NA | NA |
| FAM83C-AS1 | 2.849605 | 1.30E-27 | FAM83C antisense RNA 1 | C20orf120;NCRNA00154 | dJ614O4.3 |
| CACNA2D3-AS1 | 2.848217 | 1.54E-11 | CACNA2D3 antisense RNA 1 | NA | NA |
| ADARB2-AS1 | 2.832206 | 1.52E-11 | ADARB2 antisense RNA 1 | C10orf109;NCRNA00168 | bA466B20.1 |
| KIRREL3-AS3 | 2.755576 | 0.000219 | KIRREL3 antisense RNA 3 | PRR10;NCRNA00288 | FLJ40224 |
| CLSTN2-AS1 | 2.744559 | 5.35E-11 | CLSTN2 antisense RNA 1 | NA | NA |
| KDM4A-AS1 | 2.715113 | 1.68E-51 | KDM4A antisense RNA 1 | NA | NA |
| TBL1XR1-AS1 | 2.699156 | 2.12E-18 | TBL1XR1 antisense RNA 1 | NA | NA |
| TM4SF1-AS1 | 2.646125 | 7.26E-25 | TM4SF1 antisense RNA 1 | NA | NA |
| PAQR9-AS1 | 2.602169 | 1.43E-08 | PAQR9 antisense RNA 1 | NA | NA |
| GRM7-AS3 | 2.593783 | 1.65E-07 | GRM7 antisense RNA 3 | NA | NA |
| ST8SIA6-AS1 | 2.58617 | 5.03E-09 | ST8SIA6 antisense RNA 1 | NA | NA |
| WASIR2 | 2.568155 | 6.38E-20 | WASH and IL9R antisense RNA 2 | NCRNA00286A | NA |
| FOXC2-AS1 | 2.527077 | 5.06E-07 | FOXC2 antisense RNA 1 | NA | ODRUL |
| FGF12-AS2 | 2.520567 | 2.23E-10 | FGF12 antisense RNA 2 | NA | NA |
| MRGPRG-AS1 | 2.491277 | 0.000312 | MRGPRG antisense RNA 1 | C11orf36 | FLJ36102;HSD-40 |
| PARD3-AS1 | 2.488218 | 3.31E-28 | PARD3 antisense RNA 1 | NA | NA |
| EVX1-AS | 2.481464 | 1.67E-05 | EVX1 antisense RNA | NA | EVX1-AS1 |
| OVOL1-AS1 | 2.472796 | 1.09E-16 | OVOL1 antisense RNA 1 | NA | NA |
| TRPC7-AS1 | 2.44796 | 2.85E-07 | TRPC7 antisense RNA 1 | NA | NA |
| ANO1-AS2 | 2.447454 | 1.18E-07 | ANO1 antisense RNA 2 (head to head) | C11orf78;NCRNA00224 | NA |
| RRM1-AS1 | 2.446286 | 3.55E-12 | RRM1 antisense RNA 1 | NA | NA |
| TMPO-AS1 | 2.434768 | 1.12E-93 | TMPO antisense RNA 1 | NA | NA |
| LNX1-AS1 | 2.419178 | 8.24E-09 | LNX1 antisense RNA 1 | NA | NA |
| FRMD6-AS1 | 2.409059 | 2.83E-46 | FRMD6 antisense RNA 1 | C14orf82 | NA |
| SPATA3-AS1 | 2.404554 | 6.13E-15 | SPATA3 antisense RNA 1 (head to head) | NA | NA |
| FAM222A-AS1 | 2.398522 | 5.41E-19 | FAM222A antisense RNA 1 | NA | NA |
| KCNAB1-AS2 | 2.383457 | 3.29E-05 | KCNAB1 antisense RNA 2 | NA | NA |
| FMR1-AS1 | 2.348577 | 1.06E-13 | FMR1 antisense RNA 1 | FMR1AS;FMR1-AS | ASFMR1;FMR4 |
| DPP10-AS1 | 2.32744 | 1.88E-07 | DPP10 antisense RNA 1 | NA | NA |
| ATP2A1-AS1 | 2.321393 | 4.88E-38 | ATP2A1 antisense RNA 1 | NA | NA |
| PLCH1-AS2 | 2.317537 | 3.97E-08 | PLCH1 antisense RNA 2 | NA | NA |
| GPR50-AS1 | 2.300095 | 0.001006 | GPR50 antisense RNA 1 | NA | NA |
| CYYR1-AS1 | 2.298505 | 1.53E-12 | cysteine/tyrosine-rich 1 antisense RNA 1 | NA | NA |
| SLC7A11-AS1 | 2.288779 | 4.74E-12 | SLC7A11 antisense RNA 1 | NA | NA |
| SATB1-AS1 | 2.258575 | 9.41E-35 | SATB1 antisense RNA 1 | NA | NA |
| SIX3-AS1 | 2.248602 | 9.28E-06 | SIX3 antisense RNA 1 | NA | NA |
| MTUS2-AS1 | 2.235991 | 1.43E-10 | MTUS2 antisense RNA 1 | NA | NA |
| SAMD12-AS1 | 2.235349 | 9.25E-20 | SAMD12 antisense RNA 1 | C8orf26;NCRNA00252 | NA |
| GPR1-AS | 2.233544 | 4.55E-05 | GPR1 antisense RNA | GPR1-AS1 | GPR1AS |
| WNT5A-AS1 | 2.224305 | 3.42E-23 | WNT5A antisense RNA 1 | NA | NA |
| STPG2-AS1 | 2.198351 | 7.03E-06 | STPG2 antisense RNA 1 | NA | NA |
| MYHAS | 2.190106 | 3.18E-15 | myosin heavy chain gene cluster antisense RNA | NA | NA |
| DDR1-AS1 | 2.17198 | 2.34E-10 | DDR1 antisense RNA 1 (head to head) | TIGD1L | bPG70P20.2;bCX111D4.7;bQB10J12.1 |
| STEAP3-AS1 | 2.164034 | 2.68E-20 | STEAP3 antisense RNA 1 | NA | NA |
| DARS-AS1 | 2.161981 | 5.62E-64 | DARS antisense RNA 1 | NA | NA |
| PTGES2-AS1 | 2.161286 | 2.58E-19 | PTGES2 antisense RNA 1 (head to head) | NA | NA |
| IGSF11-AS1 | 2.155313 | 1.70E-05 | IGSF11 antisense RNA 1 | NA | NA |
| C3orf67-AS1 | 2.154421 | 5.69E-07 | C3orf67 antisense RNA 1 | NA | NA |
| CLDN10-AS1 | 2.149928 | 0.000372 | CLDN10 antisense RNA 1 | NA | NA |
| FTCD-AS1 | 2.134853 | 0.000153 | FTCD antisense RNA 1 | NA | NA |
| MCHR2-AS1 | 2.112152 | 2.43E-05 | MCHR2 antisense RNA 1 | NA | NA |
| LEF1-AS1 | 2.097331 | 1.96E-35 | LEF1 antisense RNA 1 | NA | NA |
| HMMR-AS1 | 2.096129 | 1.52E-08 | HMMR antisense RNA 1 | NA | NA |
| LARS2-AS1 | 2.066703 | 2.01E-09 | LARS2 antisense RNA 1 | NA | NA |
| UPK1A-AS1 | 2.03836 | 1.91E-06 | UPK1A antisense RNA 1 | NA | NA |
| UNC5B-AS1 | 2.036737 | 2.44E-17 | UNC5B antisense RNA 1 | NA | NA |
| PKIA-AS1 | 2.025277 | 4.58E-14 | PKIA antisense RNA 1 | NA | NA |
| LEMD1-AS1 | 2.021881 | 3.40E-13 | LEMD1 antisense RNA 1 | NA | NA |
| DLG1-AS1 | 2.010888 | 4.16E-22 | DLG1 antisense RNA 1 | NA | NA |
| YEATS2-AS1 | 1.982807 | 1.18E-31 | YEATS2 antisense RNA 1 | NA | NA |
| GRM7-AS2 | 1.982056 | 2.05E-05 | GRM7 antisense RNA 2 | NA | NA |
| EPHA5-AS1 | 1.979342 | 0.000401 | EPHA5 antisense RNA 1 | NA | NA |
| CHL1-AS1 | 1.977601 | 4.82E-08 | CHL1 antisense RNA 1 | NA | NA |
| SAMSN1-AS1 | 1.977308 | 1.40E-08 | SAMSN1 antisense RNA 1 | NA | NA |
| DGUOK-AS1 | 1.94977 | 1.54E-40 | DGUOK antisense RNA 1 | NA | NA |
| SMAD5-AS1 | 1.914734 | 1.83E-15 | SMAD5 antisense RNA 1 | SMAD5OS | DAMS |
| TSPEAR-AS2 | 1.892638 | 2.20E-09 | TSPEAR antisense RNA 2 | C21orf90 | NA |
| SIAH2-AS1 | 1.880898 | 2.35E-17 | SIAH2 antisense RNA 1 | NA | NA |
| TM4SF19-AS1 | 1.876059 | 1.87E-22 | TM4SF19 antisense RNA 1 | NA | NA |
| B4GALT4-AS1 | 1.85735 | 6.81E-07 | B4GALT4 antisense RNA 1 | NA | NA |
| LOXL1-AS1 | 1.85188 | 3.71E-40 | LOXL1 antisense RNA 1 | NA | NA |
| IGF2-AS | 1.84841 | 7.80E-07 | IGF2 antisense RNA | IGF2AS | PEG8;IGF2-AS1 |
| KLHL7-AS1 | 1.837207 | 3.71E-21 | KLHL7 antisense RNA 1 (head to head) | NA | PLATAK |
| FREM2-AS1 | 1.836377 | 0.00053 | FREM2 antisense RNA 1 | NA | NA |
| RNF217-AS1 | 1.80774 | 7.51E-20 | RNF217 antisense RNA 1 (head to head) | NA | STL |
| KTN1-AS1 | 1.796208 | 4.68E-47 | KTN1 antisense RNA 1 | C14orf33 | NA |
| CERS3-AS1 | 1.794748 | 1.74E-13 | CERS3 antisense RNA 1 | NA | NA |
| NCBP2-AS1 | 1.783992 | 4.15E-22 | NCBP2 antisense RNA 1 | NA | NA |
| RNF144A-AS1 | 1.78289 | 3.46E-17 | RNF144A antisense RNA 1 | NA | NA |
| C5orf66-AS2 | 1.775998 | 0.000393 | C5orf66 antisense RNA 2 | NA | NA |
| DENND5B-AS1 | 1.773265 | 9.90E-10 | DENND5B antisense RNA 1 | NA | NA |
| MAGEA8-AS1 | 1.771819 | 1.12E-06 | MAGEA8 antisense RNA 1 (head to head) | NA | RP5-869M20.2 |
| LNX1-AS2 | 1.751801 | 1.53E-08 | LNX1 antisense RNA 2 | NA | NA |
| PRC1-AS1 | 1.751352 | 1.85E-23 | PRC1 antisense RNA 1 | NA | NA |
| MLIP-AS1 | 1.747507 | 0.000241 | MLIP antisense RNA 1 | NA | NA |
| ARAP1-AS1 | 1.747133 | 4.47E-05 | ARAP1 antisense RNA 1 | NA | NA |
| CHRM3-AS1 | 1.745239 | 3.21E-06 | CHRM3 antisense RNA 1 | NA | NA |
| MYB-AS1 | 1.738703 | 0.001546 | MYB antisense RNA 1 | MYBAS;MYB-AS | RP1-32B1.3;NCRNA00209 |
| GATA3-AS1 | 1.726463 | 1.84E-06 | GATA3 antisense RNA 1 | NA | NA |
| MAFG-AS1 | 1.692193 | 4.65E-33 | MAFG antisense RNA 1 (head to head) | NA | NA |
| ISX-AS1 | 1.690903 | 0.002683 | ISX antisense RNA 1 | NA | NA |
| HPN-AS1 | 1.690282 | 1.95E-10 | HPN antisense RNA 1 | NA | NA |
| CLRN1-AS1 | 1.687079 | 1.24E-05 | CLRN1 antisense RNA 1 | CLRN1OS | UCRP |
| CASK-AS1 | 1.682641 | 1.95E-13 | CASK antisense RNA 1 | NA | NA |
| WASIR1 | 1.680868 | 0.003454 | WASH and IL9R antisense RNA 1 | NCRNA00286B | NA |
| ZNF252P-AS1 | 1.66411 | 4.69E-23 | ZNF252P antisense RNA 1 | C8orf77 | NA |
| TMEM147-AS1 | 1.646076 | 6.29E-27 | TMEM147 antisense RNA 1 | NA | NA |
| LAMP5-AS1 | 1.645079 | 0.000322 | LAMP5 antisense RNA 1 | NA | NA |
| DLEU7-AS1 | 1.639871 | 7.50E-20 | DLEU7 antisense RNA 1 | NA | NA |
| BRWD1-AS1 | 1.637655 | 8.15E-07 | BRWD1 antisense RNA 1 | NA | NA |
| RFPL1S | 1.635315 | 1.08E-08 | RFPL1 antisense RNA 1 | RFPL1-AS1 | RFPL1-AS;NCRNA00006 |
| GRM7-AS1 | 1.620419 | 0.000979 | GRM7 antisense RNA 1 | NA | NA |
| PCSK6-AS1 | 1.613831 | 0.008655 | PCSK6 antisense RNA 1 | NA | NA |
| DIRC3-AS1 | 1.599177 | 0.000395 | DIRC3 antisense RNA 1 | NA | NA |
| LMCD1-AS1 | 1.598692 | 4.03E-25 | LMCD1 antisense RNA 1 (head to head) | NA | NA |
| DLGAP1-AS2 | 1.585916 | 4.73E-17 | DLGAP1 antisense RNA 2 | NA | MGC11082 |
| TMEM246-AS1 | 1.585289 | 1.33E-06 | TMEM246 antisense RNA 1 | NA | NA |
| TMEM44-AS1 | 1.573958 | 4.75E-28 | TMEM44 antisense RNA 1 | NA | NA |
| MEIS1-AS3 | 1.567467 | 1.02E-06 | MEIS1 antisense RNA 3 | NA | NA |
| EXTL3-AS1 | 1.563834 | 1.98E-16 | EXTL3 antisense RNA 1 | C8orf50 | NA |
| GTSE1-AS1 | 1.557919 | 7.84E-20 | GTSE1 antisense RNA 1 (head to head) | NA | NA |
| CSE1L-AS1 | 1.540665 | 8.82E-09 | CSE1L antisense RNA 1 | NA | NA |
| CRYM-AS1 | 1.537323 | 5.08E-21 | CRYM antisense RNA 1 | NCRNA00169 | FLJ41766 |
| GNAS-AS1 | 1.536586 | 2.21E-09 | GNAS antisense RNA 1 | GNASAS;GNAS-AS | SANG;NESP-AS;NESPAS;GNAS1AS;NCRNA00075 |
| DDX39B-AS1 | 1.534049 | 2.27E-11 | DDX39B antisense RNA 1 | NA | NA |
| PRRT3-AS1 | 1.526518 | 2.27E-19 | PRRT3 antisense RNA 1 | NA | NA |
| EGFLAM-AS4 | 1.52556 | 0.026664 | EGFLAM antisense RNA 4 | NA | NA |
| UCHL1-AS1 | 1.519701 | 0.000591 | UCHL1 antisense RNA 1 (head to head) | NA | NA |
| PLCXD2-AS1 | 1.518068 | 1.66E-05 | PLCXD2 antisense RNA 1 | NA | NA |
| SBF2-AS1 | 1.512081 | 3.56E-27 | SBF2 antisense RNA 1 | NA | NA |
| HIF1A-AS1 | 1.511413 | 1.19E-12 | HIF1A antisense RNA 1 | NA | 5'aHIF-1A |
| CHKB-AS1 | 1.498412 | 4.34E-28 | CHKB antisense RNA 1 (head to head) | NA | NA |
| STAU2-AS1 | 1.476954 | 7.37E-12 | STAU2 antisense RNA 1 | NA | NA |
| NUP50-AS1 | 1.465397 | 3.09E-26 | NUP50 antisense RNA 1 (head to head) | NA | NA |
| UBE2Q1-AS1 | 1.464485 | 2.30E-16 | UBE2Q1 antisense RNA 1 | NA | NA |
| PRRX2-AS1 | 1.463055 | 0.000392 | PRRX2 antisense RNA 1 | NA | NA |
| AGAP2-AS1 | 1.461514 | 1.64E-32 | AGAP2 antisense RNA 1 | NA | LOC100130776;PUNISHER |
| MSC-AS1 | 1.452064 | 2.02E-12 | MSC antisense RNA 1 | NA | NA |
| KCTD21-AS1 | 1.451183 | 1.95E-17 | KCTD21 antisense RNA 1 | NA | NA |
| GCC2-AS1 | 1.447571 | 2.41E-28 | GCC2 antisense RNA 1 | NA | NA |
| ZBTB46-AS1 | 1.446653 | 0.025467 | ZBTB46 antisense RNA 1 | NA | NA |
| RBM12B-AS1 | 1.439339 | 5.92E-25 | RBM12B antisense RNA 1 | C8orf39 | PRO1905 |
| VAC14-AS1 | 1.436932 | 1.62E-16 | VAC14 antisense RNA 1 | NA | NA |
| NCK1-AS1 | 1.434793 | 1.02E-28 | NCK1 antisense RNA 1 (head to head) | NA | SLC35G2-AS1 |
| AADACL2-AS1 | 1.425327 | 1.67E-05 | AADACL2 antisense RNA 1 | NA | NA |
| SLC16A1-AS1 | 1.423062 | 1.05E-31 | SLC16A1 antisense RNA 1 | NA | NA |
| LMLN-AS1 | 1.421255 | 0.000401 | LMLN antisense RNA 1 | NA | NA |
| PTPRG-AS1 | 1.408887 | 3.42E-15 | PTPRG antisense RNA 1 | NA | NA |
| UBAC2-AS1 | 1.408361 | 5.48E-25 | UBAC2 antisense RNA 1 | NA | NA |
| DLGAP1-AS4 | 1.407196 | 0.005437 | DLGAP1 antisense RNA 4 | NA | NA |
| FZD10-AS1 | 1.392726 | 6.73E-09 | FZD10 antisense RNA 1 (head to head) | NA | FLJ31485 |
| MIS18A-AS1 | 1.382942 | 1.18E-15 | MIS18A antisense RNA 1 | NA | NA |
| GTF3C2-AS1 | 1.378768 | 2.21E-22 | GTF3C2 antisense RNA 1 | NA | NA |
| DNAH17-AS1 | 1.378644 | 1.59E-09 | DNAH17 antisense RNA 1 | NA | NA |
| EGFR-AS1 | 1.369709 | 3.62E-07 | EGFR antisense RNA 1 | NA | NA |
| JRKL-AS1 | 1.362077 | 0.01006 | JRKL antisense RNA 1 | NA | NA |
| OPA1-AS1 | 1.358596 | 1.93E-09 | OPA1 antisense RNA 1 | NA | NA |
| STAM-AS1 | 1.353444 | 4.02E-15 | STAM antisense RNA 1 (head to head) | NA | locus3182 |
| MCM8-AS1 | 1.339588 | 1.42E-06 | MCM8 antisense RNA 1 | NA | NA |
| FOXD2-AS1 | 1.338609 | 3.75E-25 | FOXD2 antisense RNA 1 (head to head) | NA | MGC12982 |
| PITPNA-AS1 | 1.338258 | 1.26E-32 | PITPNA antisense RNA 1 | NA | NA |
| SNCA-AS1 | 1.337041 | 0.0012 | SNCA antisense RNA 1 | NA | NA |
| STK4-AS1 | 1.324131 | 1.23E-18 | STK4 antisense RNA 1 (head to head) | NA | NA |
| RHPN1-AS1 | 1.321561 | 3.46E-15 | RHPN1 antisense RNA 1 (head to head) | C8orf51 | MGC3113 |
| RNF139-AS1 | 1.317118 | 8.89E-24 | RNF139 antisense RNA 1 (head to head) | NA | NA |
| THSD4-AS1 | 1.312202 | 0.000964 | THSD4 antisense RNA 1 | NA | NA |
| C9orf173-AS1 | 1.311325 | 3.61E-08 | C9orf173 antisense RNA 1 | NA | NA |
| FER1L6-AS1 | 1.29836 | 0.031647 | FER1L6 antisense RNA 1 | C8orf54 | FLJ35721 |
| SUCLA2-AS1 | 1.290746 | 1.97E-18 | SUCLA2 antisense RNA 1 | NA | NA |
| MCCC1-AS1 | 1.290593 | 4.39E-10 | MCCC1 antisense RNA 1 | NA | NA |
| PRKX-AS1 | 1.289 | 0.000138 | PRKX antisense RNA 1 | NA | NA |
| SYNPR-AS1 | 1.280031 | 6.12E-08 | SYNPR antisense RNA 1 | NA | NA |
| ZNF503-AS2 | 1.279319 | 2.14E-23 | ZNF503 antisense RNA 2 | C10orf41;NCRNA00245 | NA |
| DIAPH2-AS1 | 1.274064 | 1.28E-07 | DIAPH2 antisense RNA 1 | NA | NA |
| CSTF3-AS1 | 1.262635 | 1.12E-09 | CSTF3 antisense RNA 1 (head to head) | NA | NA |
| STK24-AS1 | 1.259862 | 3.78E-13 | STK24 antisense RNA 1 | NA | NA |
| BRWD1-AS2 | 1.255216 | 3.12E-16 | BRWD1 antisense RNA 2 | C21orf87;NCRNA00257;BRWD1-IT2 | NA |
| BSN-AS2 | 1.252011 | 1.49E-05 | BSN antisense RNA 2 (head to head) | NA | NA |
| ITGB5-AS1 | 1.251926 | 0.000338 | ITGB5 antisense RNA 1 | NA | NA |
| HAND2-AS1 | 1.244754 | 0.005507 | HAND2 antisense RNA 1 (head to head) | NA | DEIN;NBLA00301;FLJ11539 |
| LGALS8-AS1 | 1.23851 | 1.23E-11 | LGALS8 antisense RNA 1 | NA | NA |
| CLYBL-AS2 | 1.230207 | 0.001208 | CLYBL antisense RNA 2 | NA | NA |
| RALY-AS1 | 1.230083 | 6.49E-29 | RALY antisense RNA 1 | NA | NA |
| SNAP25-AS1 | 1.226289 | 0.000111 | SNAP25 antisense RNA 1 | NA | NA |
| COL18A1-AS2 | 1.224267 | 0.000467 | COL18A1 antisense RNA 2 | NA | NA |
| SYP-AS1 | 1.218805 | 0.002896 | SYP antisense RNA 1 | NA | NA |
| CEP83-AS1 | 1.209716 | 7.24E-14 | CEP83 antisense RNA 1 (head to head) | CCDC41-AS1 | NA |
| ZNF793-AS1 | 1.201114 | 1.78E-08 | ZNF793 antisense RNA 1 (head to head) | NA | NA |
| JHDM1D-AS1 | 1.185665 | 2.46E-15 | JHDM1D antisense RNA 1 (head to head) | NA | NA |
| BVES-AS1 | 1.182424 | 9.56E-05 | BVES antisense RNA 1 | C6orf112 | bA99L11.2 |
| WT1-AS | 1.180926 | 0.0001 | WT1 antisense RNA | WIT1 | WIT-1;WT1AS;WT1-AS1 |
| TAF1A-AS1 | 1.173654 | 6.10E-21 | TAF1A antisense RNA 1 | NA | NA |
| EMX2OS | 1.16633 | 0.00492 | EMX2 opposite strand/antisense RNA | NA | NCRNA00045;EMX2-AS1 |
| JAKMIP2-AS1 | 1.158367 | 0.001975 | JAKMIP2 antisense RNA 1 | NA | NA |
| PROX1-AS1 | 1.15831 | 0.000276 | PROX1 antisense RNA 1 | NA | NA |
| DLG5-AS1 | 1.151012 | 1.71E-15 | DLG5 antisense RNA 1 | NA | NA |
| EHMT2-AS1 | 1.148444 | 1.50E-05 | EHMT2 antisense RNA 1 | NA | NA |
| TNKS2-AS1 | 1.144432 | 2.71E-10 | TNKS2 antisense RNA 1 (head to head) | NA | NA |
| BFSP2-AS1 | 1.139337 | 0.000387 | BFSP2 antisense RNA 1 | NA | MGC2848 |
| DSG2-AS1 | 1.132328 | 3.97E-10 | DSG2 antisense RNA 1 | NA | NA |
| TSPEAR-AS1 | 1.129512 | 0.000353 | TSPEAR antisense RNA 1 | NA | NA |
| ALDH1L1-AS2 | 1.127281 | 0.001731 | ALDH1L1 antisense RNA 2 | NA | NA |
| CAPN10-AS1 | 1.121311 | 3.08E-18 | CAPN10 antisense RNA 1 (head to head) | NA | locus959 |
| ZFX-AS1 | 1.113936 | 0.000698 | ZFX antisense RNA 1 | NA | NA |
| FLG-AS1 | 1.105513 | 2.52E-05 | FLG antisense RNA 1 | NA | NA |
| POT1-AS1 | 1.097138 | 5.02E-11 | POT1 antisense RNA 1 | NA | NA |
| CCDC37-AS1 | 1.096196 | 0.034463 | CCDC37 antisense RNA 1 (head to head) | NA | NA |
| CECR5-AS1 | 1.090736 | 1.76E-09 | CECR5 antisense RNA 1 | CECR4 | NCRNA00017 |
| SNAP47-AS1 | 1.089231 | 3.91E-05 | SNAP47 antisense RNA 1 | SNAP47-IT1 | NA |
| MNX1-AS2 | 1.08811 | 0.001343 | MNX1 antisense RNA 2 | NA | NA |
| ZNF436-AS1 | 1.070127 | 2.64E-17 | ZNF436 antisense RNA 1 | C1orf213 | FLJ90508 |
| ACTN1-AS1 | 1.069206 | 4.01E-07 | ACTN1 antisense RNA 1 | C14orf84 | NA |
| VLDLR-AS1 | 1.069161 | 4.17E-06 | VLDLR antisense RNA 1 | NA | NA |
| TMEM254-AS1 | 1.068617 | 8.00E-12 | TMEM254 antisense RNA 1 | NA | NA |
| CPEB2-AS1 | 1.066935 | 1.16E-05 | CPEB2 antisense RNA 1 (head to head) | NA | NA |
| CERS6-AS1 | 1.06614 | 2.13E-05 | CERS6 antisense RNA 1 | NA | NA |
| PIK3CD-AS2 | 1.066063 | 2.80E-10 | PIK3CD antisense RNA 2 | NA | NA |
| PRMT5-AS1 | 1.056466 | 6.72E-12 | PRMT5 antisense RNA 1 | NA | NA |
| TIPARP-AS1 | 1.055228 | 7.64E-11 | TIPARP antisense RNA 1 | NA | NA |
| SLC25A25-AS1 | 1.049641 | 5.03E-10 | SLC25A25 antisense RNA 1 | NA | NA |
| RDH10-AS1 | 1.047268 | 2.85E-06 | RDH10 antisense RNA 1 | NA | NA |
| A2ML1-AS1 | 1.046389 | 0.049174 | A2ML1 antisense RNA 1 | NA | NA |
| DNAJC9-AS1 | 1.046348 | 1.60E-15 | DNAJC9 antisense RNA 1 | C10orf103 | bA537A6.3 |
| KCNK15-AS1 | 1.040722 | 7.91E-05 | KCNK15 antisense RNA 1 (head to head) | NA | RP11-445H22.4 |
| DLEU1-AS1 | 1.035138 | 0.004157 | DLEU1 antisense RNA 1 | NA | LINC01308 |
| EFCAB6-AS1 | 1.027476 | 0.000629 | EFCAB6 antisense RNA 1 | NA | CITF22-123F2.1 |
| FRMD6-AS2 | 1.017927 | 0.005142 | FRMD6 antisense RNA 2 | NA | NA |
| DLGAP1-AS5 | 1.016197 | 0.022638 | DLGAP1 antisense RNA 5 | NA | NA |
| MYLK-AS1 | 1.005269 | 1.40E-11 | MYLK antisense RNA 1 | NA | NA |
| PIK3CD-AS1 | -1.03498 | 1.01E-05 | PIK3CD antisense RNA 1 | C1orf200 | NA |
| SHANK2-AS2 | -1.03845 | 0.003391 | SHANK2 antisense RNA 2 | NA | NA |
| ITPK1-AS1 | -1.04626 | 0.000192 | ITPK1 antisense RNA 1 | C14orf85;NCRNA00203;ITPK1AS;ITPK1-AS | NA |
| DENND6A-AS1 | -1.06825 | 5.67E-09 | DENND6A antisense RNA 1 | NA | NA |
| CARD8-AS1 | -1.0713 | 1.22E-14 | CARD8 antisense RNA 1 | NA | LOC100505812 |
| DNMBP-AS1 | -1.07308 | 4.90E-10 | DNMBP antisense RNA 1 | NCRNA00093 | FLJ40792;bA287G8.2 |
| C21orf62-AS1 | -1.07341 | 6.21E-15 | C21orf62 antisense RNA 1 | C21orf49 | NA |
| WWC3-AS1 | -1.0767 | 7.31E-05 | WWC3 antisense RNA 1 | NA | NA |
| PCED1B-AS1 | -1.07925 | 9.11E-10 | PCED1B antisense RNA 1 | NA | NA |
| DOCK4-AS1 | -1.08283 | 2.55E-05 | DOCK4 antisense RNA 1 | NA | NA |
| MBNL1-AS1 | -1.08934 | 3.59E-12 | MBNL1 antisense RNA 1 | NA | NA |
| COL18A1-AS1 | -1.09614 | 0.000327 | COL18A1 antisense RNA 1 | C21orf123;NCRNA00175 | PRED80 |
| SUCLG2-AS1 | -1.11107 | 3.36E-20 | SUCLG2 antisense RNA 1 (head to head) | NA | NA |
| ZNF503-AS1 | -1.11534 | 4.43E-05 | ZNF503 antisense RNA 1 | NA | NA |
| TRG-AS1 | -1.11971 | 2.00E-10 | T cell receptor gamma locus antisense RNA 1 | NA | NA |
| CELF2-AS1 | -1.13163 | 2.36E-05 | CELF2 antisense RNA 1 | C10orf31 | FLJ40494;Em:AC026887.2 |
| UCKL1-AS1 | -1.13722 | 3.43E-10 | UCKL1 antisense RNA 1 | UCKL1OS;UCKL1AS;UCKL1-AS | FLJ46647 |
| BOK-AS1 | -1.13779 | 0.00279 | BOK antisense RNA 1 | BOKAS | NCRNA00151;NAToB |
| SRGAP3-AS4 | -1.14275 | 0.001119 | SRGAP3 antisense RNA 4 | NA | NA |
| PKN2-AS1 | -1.15134 | 7.13E-10 | PKN2 antisense RNA 1 | NA | NA |
| ITIH4-AS1 | -1.1631 | 2.44E-05 | ITIH4 antisense RNA 1 | NA | NA |
| MAGI1-AS1 | -1.1866 | 0.020596 | MAGI1 antisense RNA 1 | NA | NA |
| AQP4-AS1 | -1.20561 | 0.000113 | AQP4 antisense RNA 1 | C18orf16;CHST9-AS1 | FLJ30507 |
| WDR11-AS1 | -1.20605 | 1.25E-06 | WDR11 antisense RNA 1 | NA | NA |
| SMARCA5-AS1 | -1.20873 | 1.00E-08 | SMARCA5 antisense RNA 1 | NA | NA |
| SH3BP5-AS1 | -1.22052 | 1.12E-16 | SH3BP5 antisense RNA 1 | NA | NA |
| PSMD6-AS2 | -1.23412 | 5.94E-17 | PSMD6 antisense RNA 2 | NA | NA |
| SH3RF3-AS1 | -1.23748 | 2.89E-15 | SH3RF3 antisense RNA 1 | NA | NA |
| TMEM5-AS1 | -1.24723 | 6.75E-07 | TMEM5 antisense RNA 1 | NA | NA |
| EHD4-AS1 | -1.25402 | 6.39E-13 | EHD4 antisense RNA 1 | NA | NA |
| SMC2-AS1 | -1.2575 | 8.85E-11 | SMC2 antisense RNA 1 (head to head) | NA | NA |
| NALCN-AS1 | -1.26027 | 9.24E-06 | NALCN antisense RNA 1 | NA | NA |
| NEXN-AS1 | -1.27249 | 2.39E-16 | NEXN antisense RNA 1 | C1orf118 | FLJ90637 |
| C1QTNF1-AS1 | -1.2727 | 0.000124 | C1QTNF1 antisense RNA 1 | NA | NA |
| JAZF1-AS1 | -1.27581 | 1.15E-10 | JAZF1 antisense RNA 1 | NA | NA |
| CPB2-AS1 | -1.28677 | 1.28E-07 | CPB2 antisense RNA 1 | NA | NA |
| KRTAP5-AS1 | -1.29274 | 4.74E-07 | KRTAP5-1/KRTAP5-2 antisense RNA 1 | NA | NA |
| CATIP-AS2 | -1.29508 | 3.09E-06 | CATIP antisense RNA 2 | NA | NA |
| ST7-AS2 | -1.29893 | 1.40E-08 | ST7 antisense RNA 2 | ST7OT2 | ST7AS2 |
| IL10RB-AS1 | -1.30087 | 3.85E-34 | IL10RB antisense RNA 1 (head to head) | NA | IFNAR2-AS1 |
| VIM-AS1 | -1.30566 | 3.35E-14 | VIM antisense RNA 1 | NA | NA |
| NCOA7-AS1 | -1.3083 | 8.09E-05 | NCOA7 antisense RNA 1 | NA | NA |
| FAM170B-AS1 | -1.31031 | 0.024222 | FAM170B antisense RNA 1 | NA | NA |
| BAIAP2-AS1 | -1.32098 | 1.98E-34 | BAIAP2 antisense RNA 1 (head to head) | NA | NA |
| FGF13-AS1 | -1.32551 | 4.24E-05 | FGF13 antisense RNA 1 | NA | NA |
| EPB41L4A-AS2 | -1.33739 | 7.43E-26 | EPB41L4A antisense RNA 2 (head to head) | NA | FLJ11235 |
| GLTSCR1-AS1 | -1.34579 | 1.30E-05 | GLTSCR1 antisense RNA 1 | NA | NA |
| PAXIP1-AS2 | -1.35959 | 2.26E-47 | PAXIP1 antisense RNA 2 | PAXIP1OS | NA |
| LMF1-AS1 | -1.36204 | 4.01E-08 | LMF1 antisense RNA 1 | NA | NA |
| SPIN4-AS1 | -1.3694 | 2.04E-05 | SPIN4 antisense RNA 1 | NA | NA |
| TRHDE-AS1 | -1.37721 | 0.003186 | TRHDE antisense RNA 1 | NA | NA |
| PSMG3-AS1 | -1.3821 | 3.24E-22 | PSMG3 antisense RNA 1 (head to head) | NA | KIAA1908 |
| TTLL10-AS1 | -1.38414 | 2.23E-06 | TTLL10 antisense RNA 1 | NA | NA |
| FOXP1-AS1 | -1.38613 | 3.46E-13 | FOXP1 antisense RNA 1 | NA | NA |
| ADPGK-AS1 | -1.39552 | 1.07E-22 | ADPGK antisense RNA 1 | NA | NA |
| DIO3OS | -1.4572 | 8.79E-09 | DIO3 opposite strand/antisense RNA (head to head) | C14orf134 | NCRNA00041;DIO3-AS1 |
| HNF1A-AS1 | -1.45997 | 2.07E-06 | HNF1A antisense RNA 1 | C12orf27;NCRNA00262 | FLJ38690 |
| TMEM72-AS1 | -1.46315 | 3.78E-15 | TMEM72 antisense RNA 1 | NA | NA |
| SHANK2-AS1 | -1.46772 | 5.01E-06 | SHANK2 antisense RNA 1 | NA | NA |
| VAV3-AS1 | -1.46823 | 2.35E-05 | VAV3 antisense RNA 1 | NA | NA |
| HLA-DQB1-AS1 | -1.47021 | 1.03E-11 | HLA-DQB1 antisense RNA 1 | NA | NA |
| C2-AS1 | -1.49248 | 1.31E-13 | C2 antisense RNA 1 | NA | NA |
| ABHD11-AS1 | -1.49366 | 9.11E-13 | ABHD11 antisense RNA 1 (tail to tail) | WBSCR26;LINC00035 | NCRNA00035 |
| CFLAR-AS1 | -1.50001 | 1.35E-21 | CFLAR antisense RNA 1 | ALS2CR10 | NA |
| ZNF582-AS1 | -1.50931 | 6.12E-18 | ZNF582 antisense RNA 1 (head to head) | NA | NA |
| BTBD9-AS1 | -1.51855 | 3.26E-07 | BTBD9 antisense RNA 1 | NA | NA |
| USP30-AS1 | -1.55344 | 1.07E-16 | USP30 antisense RNA 1 | NA | NA |
| ZBTB20-AS4 | -1.58036 | 1.93E-11 | ZBTB20 antisense RNA 4 | NA | NA |
| GRPEL2-AS1 | -1.60149 | 1.18E-18 | GRPEL2 antisense RNA 1 | NA | RP11-394O4.4 |
| RARA-AS1 | -1.61799 | 8.98E-52 | RARA antisense RNA 1 | NA | NA |
| SLC6A1-AS1 | -1.6257 | 3.20E-09 | SLC6A1 antisense RNA 1 | NA | NA |
| WDR86-AS1 | -1.63078 | 8.68E-10 | WDR86 antisense RNA 1 | NA | NA |
| TBX2-AS1 | -1.63367 | 3.14E-16 | TBX2 antisense RNA 1 | NA | NA |
| WWC2-AS2 | -1.64623 | 1.79E-23 | WWC2 antisense RNA 2 | C4orf38 | FLJ30277 |
| RBMS3-AS3 | -1.65239 | 1.31E-13 | RBMS3 antisense RNA 3 | NA | NA |
| ARMC2-AS1 | -1.66694 | 3.04E-09 | ARMC2 antisense RNA 1 | NA | NA |
| MRGPRF-AS1 | -1.68142 | 6.55E-12 | MRGPRF antisense RNA 1 | NA | NA |
| EP300-AS1 | -1.68546 | 1.02E-32 | EP300 antisense RNA 1 | NA | NA |
| LY86-AS1 | -1.6874 | 1.36E-10 | LY86 antisense RNA 1 | LY86AS;LY86-AS | FLJ33708 |
| PRR29-AS1 | -1.70138 | 5.64E-12 | PRR29 antisense RNA 1 | NA | NA |
| OVCH1-AS1 | -1.70243 | 1.15E-08 | OVCH1 antisense RNA 1 | NA | NA |
| HORMAD2-AS1 | -1.70531 | 5.92E-10 | HORMAD2 antisense RNA 1 | NA | MTMR3-AS1;NONHSAG033653 |
| TAT-AS1 | -1.7114 | 1.21E-12 | TAT antisense RNA 1 | NA | NA |
| PRICKLE2-AS3 | -1.71413 | 6.65E-12 | PRICKLE2 antisense RNA 3 | NA | NA |
| SEMA3B-AS1 | -1.72238 | 1.40E-21 | SEMA3B antisense RNA 1 (head to head) | NA | NA |
| NR2F2-AS1 | -1.72393 | 1.64E-25 | NR2F2 antisense RNA 1 | NA | NA |
| TMEM220-AS1 | -1.72751 | 1.53E-33 | TMEM220 antisense RNA 1 | NA | NA |
| NFIA-AS2 | -1.75409 | 1.12E-12 | NFIA antisense RNA 2 | NA | NA |
| WDFY3-AS2 | -1.76715 | 5.88E-46 | WDFY3 antisense RNA 2 | C4orf12;NCRNA00247 | FBI4 |
| ZRANB2-AS1 | -1.79975 | 9.22E-25 | ZRANB2 antisense RNA 1 | NA | NA |
| CYP1B1-AS1 | -1.81755 | 1.19E-25 | CYP1B1 antisense RNA 1 | C2orf58 | MGC34824 |
| GPC5-AS1 | -1.82437 | 0.002556 | GPC5 antisense RNA 1 | NA | NA |
| COL4A2-AS1 | -1.83505 | 2.22E-18 | COL4A2 antisense RNA 1 | NA | NA |
| CCDC13-AS1 | -1.85297 | 5.64E-25 | CCDC13 antisense RNA 1 | NA | NA |
| ST3GAL5-AS1 | -1.86149 | 1.97E-22 | ST3GAL5 antisense RNA 1 (head to head) | NA | NA |
| PRICKLE2-AS1 | -1.86658 | 2.04E-15 | PRICKLE2 antisense RNA 1 | NA | NA |
| KLHL30-AS1 | -1.91841 | 1.10E-13 | KLHL30 antisense RNA 1 | C2orf19 | NA |
| MAMDC2-AS1 | -1.9404 | 2.10E-34 | MAMDC2 antisense RNA 1 | NA | NA |
| CLYBL-AS1 | -1.94767 | 0.015937 | CLYBL antisense RNA 1 | NA | NA |
| MGAT3-AS1 | -1.94911 | 7.34E-09 | MGAT3 antisense RNA 1 | NA | TapSAKI |
| P4HA2-AS1 | -1.9616 | 2.48E-17 | P4HA2 antisense RNA 1 | NA | NA |
| APOA1-AS | -1.99128 | 7.47E-44 | APOA1 antisense RNA | NA | NA |
| C20orf166-AS1 | -1.99749 | 2.47E-13 | C20orf166 antisense RNA 1 | C20orf200;NCRNA00335 | FLJ30313 |
| MEOX2-AS1 | -2.03616 | 3.12E-13 | MEOX2 antisense RNA 1 (head to head) | NA | NA |
| FAM181A-AS1 | -2.04633 | 3.84E-09 | FAM181A antisense RNA 1 | C14orf86 | NA |
| KCNQ1-AS1 | -2.05349 | 5.68E-15 | KCNQ1 antisense RNA 1 | NA | NA |
| ZEB2-AS1 | -2.06958 | 5.96E-40 | ZEB2 antisense RNA 1 | ZEB2AS;ZEB2-AS | ZEB2NAT |
| ADAMTSL4-AS1 | -2.07286 | 2.92E-40 | ADAMTSL4 antisense RNA 1 | C1orf138 | FLJ45786 |
| PKNOX2-AS1 | -2.11416 | 1.74E-09 | PKNOX2 antisense RNA 1 (head to head) | NA | NA |
| ATP11A-AS1 | -2.17764 | 2.03E-11 | ATP11A antisense RNA 1 | NA | NA |
| NTM-AS1 | -2.17966 | 0.000126 | NTM antisense RNA 1 | C11orf39 | FLJ45436 |
| TTC39A-AS1 | -2.1853 | 1.43E-21 | TTC39A antisense RNA 1 | NA | NA |
| LHFPL3-AS1 | -2.19607 | 5.34E-08 | LHFPL3 antisense RNA 1 | NA | NA |
| MEF2C-AS1 | -2.23526 | 1.31E-29 | MEF2C antisense RNA 1 | NA | NA |
| PLCH1-AS1 | -2.24359 | 1.92E-15 | PLCH1 antisense RNA 1 | NA | NA |
| MAGI2-AS3 | -2.25734 | 6.99E-48 | MAGI2 antisense RNA 3 | NA | NA |
| CELF2-AS2 | -2.27264 | 0.013234 | CELF2 antisense RNA 2 | NA | NA |
| F11-AS1 | -2.29221 | 2.98E-10 | F11 antisense RNA 1 | NA | NA |
| PRICKLE2-AS2 | -2.29975 | 2.65E-14 | PRICKLE2 antisense RNA 2 | NA | NA |
| TLR8-AS1 | -2.31909 | 2.30E-11 | TLR8 antisense RNA 1 | NA | NA |
| NAV2-AS5 | -2.32883 | 2.46E-05 | NAV2 antisense RNA 5 | NA | NA |
| MED4-AS1 | -2.37075 | 1.05E-52 | MED4 antisense RNA 1 | MED4-AS | NA |
| VIPR1-AS1 | -2.45658 | 1.05E-37 | VIPR1 antisense RNA 1 | NA | NA |
| ATP13A4-AS1 | -2.46964 | 2.38E-08 | ATP13A4 antisense RNA 1 | NA | NA |
| NKX2-1-AS1 | -2.52125 | 4.29E-14 | NKX2-1 antisense RNA 1 | NA | NA |
| CNTN4-AS1 | -2.53727 | 2.77E-11 | CNTN4 antisense RNA 1 | NA | NA |
| NAV2-AS2 | -2.54198 | 3.97E-19 | NAV2 antisense RNA 2 | NA | NA |
| TARID | -2.56042 | 4.06E-21 | TCF21 antisense RNA inducing promoter demethylation | EYA4-AS1 | NA |
| SFTPD-AS1 | -2.6617 | 6.99E-30 | SFTPD antisense RNA 1 | NA | NA |
| TBX5-AS1 | -2.68383 | 4.43E-53 | TBX5 antisense RNA 1 | NA | NA |
| HHIP-AS1 | -2.69874 | 1.19E-33 | HHIP antisense RNA 1 | NA | NA |
| FGF10-AS1 | -2.73515 | 3.32E-10 | FGF10 antisense RNA 1 | NA | RP11-473L15.2 |
| SYNE1-AS1 | -2.74603 | 1.85E-21 | SYNE1 antisense RNA 1 | NA | NA |
| SLC16A12-AS1 | -2.77291 | 1.14E-09 | SLC16A12 antisense RNA 1 | NA | NA |
| HLX-AS1 | -2.81534 | 1.88E-37 | HLX antisense RNA 1 | NA | NA |
| STARD13-AS | -2.82969 | 6.98E-39 | STARD13 antisense RNA | STARD13-AS2 | NA |
| SLC14A2-AS1 | -2.83477 | 9.96E-29 | SLC14A2 antisense RNA 1 | NA | NA |
| RBPMS-AS1 | -2.86287 | 1.15E-57 | RBPMS antisense RNA 1 | NA | NA |
| GNA14-AS1 | -2.92055 | 3.63E-22 | GNA14 antisense RNA 1 | NA | NA |
| C8orf34-AS1 | -2.9615 | 2.76E-20 | C8orf34 antisense RNA 1 | NA | NA |
| PGM5P3-AS1 | -3.01048 | 6.12E-18 | PGM5P3 antisense RNA 1 | NA | FAM233B |
| UMODL1-AS1 | -3.03288 | 2.72E-17 | UMODL1 antisense RNA 1 | C21orf128 | FLJ33471 |
| MYO16-AS1 | -3.04597 | 2.09E-21 | MYO16 antisense RNA 1 | NA | NA |
| HHATL-AS1 | -3.08629 | 2.92E-15 | HHATL antisense RNA 1 | NA | NA |
| CADM3-AS1 | -3.16583 | 4.51E-28 | CADM3 antisense RNA 1 | NA | CTA-134P22.2 |
| PDZRN3-AS1 | -3.22085 | 1.09E-25 | PDZRN3 antisense RNA 1 | NA | NA |
| GATA6-AS1 | -3.22521 | 1.31E-58 | GATA6 antisense RNA 1 (head to head) | NA | locus5689 |
| TMEM108-AS1 | -3.22806 | 1.17E-21 | TMEM108 antisense RNA 1 | NA | NA |
| PGM5-AS1 | -3.30756 | 1.48E-37 | PGM5 antisense RNA 1 | NA | FAM233A |
| SRGAP3-AS2 | -3.35754 | 5.34E-15 | SRGAP3 antisense RNA 2 | NA | NA |
| PGM5P4-AS1 | -3.40056 | 2.09E-37 | PGM5P4 antisense RNA 1 | NA | FAM233C |
| ADAMTS9-AS2 | -3.58471 | 6.05E-99 | ADAMTS9 antisense RNA 2 | NA | NA |
| LDLRAD4-AS1 | -3.83322 | 6.31E-50 | LDLRAD4 antisense RNA 1 | NA | NA |
| ADAMTS9-AS1 | -3.94 | 1.98E-67 | ADAMTS9 antisense RNA 1 | NA | NA |
| HID1-AS1 | -4.06144 | 1.92E-93 | HID1 antisense RNA 1 | NA | NA |
| LANCL1-AS1 | -4.1509 | 2.15E-142 | LANCL1 antisense RNA 1 | NA | LANCLOT |
| LHFPL3-AS2 | -4.21264 | 1.94E-49 | LHFPL3 antisense RNA 2 | NA | NA |
| PACRG-AS3 | -4.4669 | 1.32E-37 | PACRG antisense RNA 3 | NA | NA |
